# Construction of Zn(II) Linear Trinuclear Secondary Building Units from A Coordination Polymer Based on α-Acetamidocinnamic Acid and 4-Phenylpyridine

**DOI:** 10.3390/molecules25163615

**Published:** 2020-08-09

**Authors:** Daniel Ejarque, Teresa Calvet, Mercè Font-Bardia, Josefina Pons

**Affiliations:** 1 Departament de Química, Universitat Autònoma de Barcelona, Bellaterra, 08193 Barcelona, Spain; daniel.ejarque@uab.cat; 2 Departament de Mineralogia, Petrologia i Geologia Aplicada, Universitat de Barcelona, Martí i Franquès s/n, 08028 Barcelona, Spain; mtcalvet@ub.edu; 3 Unitat de Difracció de Raig-X, Centres Científics i Tecnològics de la Universitat de Barcelona (CCiTUB), Universitat de Barcelona, Solé i Sabarís, 1-3, 08028 Barcelona, Spain; mercef@ccit.ub.edu

**Keywords:** Zn(II), α-acetamidocinnamic acid, coordination polymer, trinuclear complexes, secondary building unit, X-ray crystal structures, hirshfeld surface analysis, electrostatic surface potential

## Abstract

The synthesis and characterization of one coordination polymer and two trinuclear complexes are presented. The coordination polymer [Zn_2_(µ-O,O’-ACA)_2_(ACA)_2_(4-Phpy)_2_]_n_ (**1**) has been obtained by the reaction between Zn(OAc)_2_·2H_2_O, α-acetamidocinnamic acid (HACA), and 4-phenylpyridine (4-Phpy) using EtOH as solvent. Its recrystallization in CH_3_CN or EtOH yields two trinuclear complexes, both having pinwheel arrays with formulas [Zn_3_(µ-ACA)_6_(4-Phpy)_2_]·4CH_3_CN (**2**·4CH_3_CN) and [Zn_3_(µ-ACA)_6_(EtOH)_2_]·4EtOH (**3**·4EtOH), respectively. These trinuclear species, unavoidably lose their solvent co-crystallized molecules at RT yielding the complexes [Zn_3_(µ-ACA)_6_(4-Phpy)_2_] (**2**) and [Zn_3_(µ-ACA)_6_(EtOH)_2_] (**3**). In addition, compound **2** has also been obtained reacting Zn(OAc)_2_·2H_2_O, HACA, and 4-Phpy in a 1:2:2 ratio using CH_3_CN as solvent. Compounds **1**–**3** have been characterized by analytical and spectroscopic techniques. Furthermore, single crystals suitable for X-ray diffraction method for compounds **1**, **2**·4CH_3_CN, and **3**·4EtOH were obtained and their supramolecular interactions have been studied and discussed, showing 2D supramolecular planes for the trinuclear complexes and a 3D supramolecular network for the coordination polymer. Finally, the supramolecular interactions of **2**·4CH_3_CN and **3**·4EtOH have been compared using Hirshfeld surface analysis and electrostatic potential calculations.

## 1. Introduction

The synthesis of coordination polymers (CPs) and discrete polynuclear coordination complexes has become a promising research area during the last decades. The polynuclear nature of these entities, being used as secondary building units (SBUs), serve as rigid, directional, and stable building blocks. Besides, their combination with organic polytopic linkers by coordination bonds build up metal-organic frameworks (MOFs) with a broad variety of applications in catalysis [1], separation [2], gas storage [3], magnetism [4], sensing [5], and so on [6,7,8]. Moreover, their simple synthetic methodology and ability to easily join metal atoms compared with primary (mononuclear metal center) or tertiary (metal-organic polyhedron) building units have established their study as one of the main topics in MOFs research [9,10,11]. Aside from coordination bonds, supramolecular interactions (H-bonds, C-H···π, π···π interactions) are also a determining factor which influence in the final structural array of the crystalline materials through its cooperative effect [12,13]. These weak interactions are also important in the final properties of the designed materials owing to their possibility to interact with host molecules displaying interesting applications [14,15].

Among all the SBUs, those containing carboxylate ligands are of special interest for their capability to display a great variety of coordination modes, forming di-, tri-, or tetranuclear metal carboxylates [16,17]. In addition, the presence of different functional groups in these units promote the formation of patterns of supramolecular interactions known as supramolecular synthons, which also contribute to the stabilization of the crystal packing through intermolecular interactions [12]. It is noteworthy to mention that all these structural features are influenced by many factors which dictate the final structural scaffold, such as the ionic counterions, the metal/linker ratio, the temperature, or the solvent polarity [17].

Particularly, carboxylate trinuclear compounds based on pinwheel motifs with formula [M_3_(CO_2_)_6_(L)_n_] (n = 2 or 4), where the coordination number of the lateral positions could be four or five depending on the metal center, have been used as SBUs for the synthesis of MOFs showing potential catalytic [18], magnetic [19], and fluorescent [20] properties. Furthermore, the fact that a high number of these types of arrays contain labile solvent molecules on their lateral positions, makes them appropriate for the exchange of either their lateral positions or their carboxylate moieties by polytopic linkers, conserving the pinwheel array and forming 2D or 3D materials [16,21,22]. Several complexes presenting this type of structure have been reported using a large variety of metal centers [19,21,22,23,24,25,26,27,28], being the zinc containing linear trinuclear complexes one of the most employed [20,29,30,31,32,33,34].

Zinc carboxylate complexes have been extensively used due to the great variety of coordination numbers of their carboxylate ligands (from two to eight, with four, five and six being most common) and arrangements [35,36]. Moreover, presenting a d^10^ configuration, Zn(II) atoms contribute to eliminate the ligand field effect and favor the generation of diverse geometries as well as the reorganization of the ligands into highly ordered arrays, owing to its zero crystal field stabilization energy. All these characteristics together with their luminescent [37,38] and biological applications [39,40], make their use interesting as metal centers.

Recently, our group has been studying the reactivity of cinnamic acid derivative ligands towards Cu(II) [41], Zn(II), and Cd(II) [42] metal nodes. Specifically, the α-acetamidocinnamic acid (HACA), which incorporates an additional acetamide group in the cinnamic acid structure, has been examined in combination with the d^10^ metal ions Zn(II) and Cd(II) yielding isostructural monomeric complexes ([M(ACA)_2_(H_2_O)_2_], M = Zn(II) or Cd(II)), whose reactivity in presence of the 4-phenylpyridine ligand show a different behavior between Zn(II) and Cd(II), yielding a monomeric [Zn(4-Phpy)_2_(ACA)_2_(H_2_O)_2_]·3H_2_O and a dimeric [Cd(4-Phpy)_2_(μ-ACA)(ACA)]_2_·2EtOH complexes. To the best of our knowledge, the use of ACA as a ligand for the synthesis of coordination complexes has not been previously studied before this recent publication [42]. Despite that, its use for the asymmetric hydrogenation of its double bond has been tested in a few publications [43,44,45,46,47] as well as their potential use as a precursor in the formation of L-phenylalanine amino acid [48,49].

**Scheme 1:**
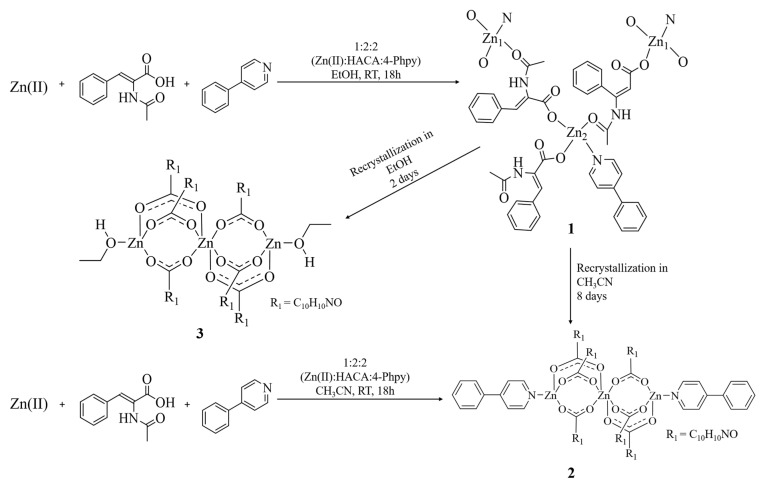
Outline of the synthetic schemes for compounds **1**–**3**.

As a continuation of our previous study, in this contribution we have assayed the reaction between Zn(OAc)_2_·2H_2_O, HACA, and 4-Phpy using EtOH as solvent, yielding the CP [Zn_2_(µ-O,O’-ACA)_2_(ACA)_2_(4-Phpy)_2_]_n_ (**1**), which by recrystallization in CH_3_CN or EtOH forms the trinuclear complexes with pinwheel arrays [Zn_3_(µ-ACA)_6_(4-Phpy)_2_]·4CH_3_CN (**2**·4CH_3_CN) and [Zn_3_(µ-ACA)_6_(EtOH)_2_]·4EtOH (**3**·4EtOH), respectively. These trinuclear species unavoidably lose their solvent co-crystallized molecules at room temperature (RT) yielding the complexes [Zn_3_(µ-ACA)_6_(4-Phpy)_2_] (**2**) and [Zn_3_(µ-ACA)_6_(EtOH)_2_] (**3**). Moreover, compound **2** was also obtained by an alternative method through the reaction between Zn(OAc)_2_·2H_2_O, HACA, and 4-Phpy using CH_3_CN as solvent at RT (Scheme 1).

## 2. Results and Discussion

### 2.1. Synthesis and Characterization

Compound **1** was prepared *via* combination of Zn(OAc)_2_·2H_2_O, HACA, and 4-Phpy in a 1:2:2 molar ratio using EtOH as solvent at room temperature (RT). When compound **1** was recrystallized in CH_3_CN or EtOH, the trinuclear species **2**·4CH_3_CN or **3**·4EtOH were obtained. The corresponding co-crystallized solvent molecules were unavoidably lost during the work-up of the powders at RT, resulting in **2** and **3**. Furthermore, compound **2** was also obtained by the reaction between Zn(OAc)_2_·2H_2_O, HACA, and 4-Phpy in a 1:2:2 molar ratio using CH_3_CN as solvent at RT (see Experimental Section, Method B). By contrast, in our previous paper [42], the complexes [Zn(4-Phpy)_2_(ACA)_2_(H_2_O)_2_]·3H_2_O and [Cd(4-Phpy)_2_(μ-ACA)(ACA)]_2_·2EtOH were obtained from the reaction between [M(ACA)_2_(H_2_O)_2_] (M = Zn(II) or Cd(II)) and 4-Phpy ligand in a 1:4 ratio for the Zn(II) complex, and a 1:2 ratio for the Cd(II) complex, using EtOH as solvent at RT.

Compounds **1**–**3** were characterized by elemental analysis, FTIR-ATR, ^1^H, ^13^C{^1^H}, and DEPT-135 NMR spectroscopies and single crystal X-ray diffraction method. Moreover, HR-ESI-MS spectrometry was carried out for **1** using CH_3_CN and EtOH as solvents. The crystal structures **2**·4CH_3_CN and **3**·4EtOH were also compared using Hirshfeld Surface Analysis and electrostatic potential calculations.

The elemental analysis of compounds **1**–**3** agrees with the proposed formula without co-crystallized solvent molecules. The positive ionization mass spectra (ESI^+^-MS) of **1** were recorded in CH_3_CN and EtOH using the same experimental conditions. In both solvents, compound **1** was broken resulting in the same monomeric fragment with *m*/*z* 495.0505 (100%) attributable to [Zn(ACA)_2_ + Na]^+^ (Figure S1a,b). Moreover, in the CH_3_CN spectrum two additional fragments were observed, one with *m*/*z* 650.1235 (100%) corresponding to [Zn(ACA)_2_(4-Phpy) + Na]^+^ and the other with *m*/*z* 967.1127 (89%) assigned to [Zn_2_(ACA)_4_ + Na]^+^ (Figure S1c,d). The FTIR-ATR spectra of **1**–**3** displayed the characteristic carboxylate bands in the range 1583–1563 cm^−1^ for ν_as_(COO), and between 1392 and 1379 cm^−1^ for ν_s_(COO). The difference between these bands [Δ = ν_as_(COO) - ν_s_(COO)] is 204 (**1**), 183 (**2**) and 171 (**3**) cm^−1^, suggesting monodentate (**1**) and bridged (**2** and **3**) coordination modes of the carboxylate moieties [50,51] (Figures S2–S4). Moreover, some specific groups of the ACA ligands such as the amine unit (ν(N-H)), the alkene moieties (ν(C-H)_alk_) or the carbonyl group (ν(C=O)), as well as the bands attributable to the aromatic groups ν(C-H)_ar_, ν(C=C/C=N), δ(C-H)_ip_, and δ(C-H)_oop_ are also identified [52]. Furthermore, the presence of coordinated EtOH molecules in **3** allows further identification of some bands between 3308 and 3275 cm^−1^ assignable to ν(O-H).

The ^1^H and ^13^C{^1^H} NMR spectra of complexes **1**–**3** have been recorded in dmso-*d*_6_ solutions showing a displacement with respect to the free ligands. In the ^1^H NMR, the signals attributable to the amine unit of the ACA ligand appeared between 9.30 and 9.19 ppm, followed by the aromatic protons of the 4-Phpy ligand, which are only observed in compounds **1** and **2** between 8.77 and 7.53 ppm owing to the absence of 4-Phpy in compound **3**. It is noteworthy that the change in the order of the *o*-*H* and the *m*-*H* atoms of the pyridyl ring from the 4-Phpy ligands between **1** and **2**, appearing at 7.91 and 7.86 for **1** and at 7.76 and 7.82 ppm for **2**. In addition, the *o*-*H* of the ACA ligand appears between 7.55 and 7.51 for **1**–**3**, and it is overlapped with some of the aromatic protons of the 4-Phpy previously mentioned in **1** and **2**, displaying a multiplet signal for those spectra. Furthermore, the remaining aromatic protons from the ACA ligand are shown between 7.36 and 7.27 ppm, being remarkable another change in the order of appearance in compound **1** respect to the trinuclear complexes **2** and **3**. In compound **1**, the alkene proton appears first at 7.30 ppm, followed by the *p*-*H* of the ACA at 7.27 ppm. By contrast, in compounds **2** and **3** the order of appearance is changed, appearing the *p*-*H* of the ACA at 7.29 ppm and the alkene protons at 7.25 (**2**) and 7.24 (**3**) ppm. The signals of the methyl protons from the ACA ligands are also observed between 1.99 and 1.95 ppm (Figures S5–S7). Furthermore, in compound **3**, apart from the signals of the ACA ligand, those corresponding to the coordinated EtOH molecules are also observed displaying three signals at 4.39, 3.45 and 1.05 corresponding to the hydroxyl moiety, the C*H*_2_ and C*H*_3_ groups, respectively.

In the ^13^C{^1^H} NMR spectra of compounds **1**–**3** the carbon atoms from the carbonyl groups appear between 170.8 and 170.6 ppm, followed by the carboxylate carbon atoms, which appear at 168.5 ppm for the three complexes. The two carbons from the alkene groups appear separately, being at 128.3 and 128.2 ppm the not protonated and at 135.1 ppm the protonated one. Then, the aromatic protons from the ACA ligand appear between 130.0 and 128.4 ppm, which required DEPT-135 experiments in order to distinguish between the *o*-*C* and the quaternary carbon of the aromatic ring (Figures S8–S10). The last atoms of the ACA ligand correspond to the methyl carbon atoms, which appear at 23.1 ppm.

The signals corresponding to the 4-Phpy ligand in **1** and **2** start at 150.0 ppm (*o*-*C* from the pyridyl ring), followed by the *p*-*C* of the same ring (149.1–148.8 ppm) and the quaternary carbon atom from the phenyl ring (136.6–136.4 ppm). The next signals from the 4-Phpy are shown at 129.4 ppm, where the *m*-*C* and *p*-*C* from the phenyl ring appear together. Finally, the *o*-*C* from the phenyl (127.2 ppm) and the *m*-*C* from the pyridyl (122.1–121.9 ppm) appear as the last signals from the 4-Phpy ligand. In addition, in compound **3**, the signals corresponding to the coordinated EtOH molecules appeared at 56.1 and 18.6 ppm, corresponding to the *C*H_2_ and *C*H_3_ carbon atoms, respectively.

### 2.2. Crystal and Extended Structure of **1**

**Figure 1:**
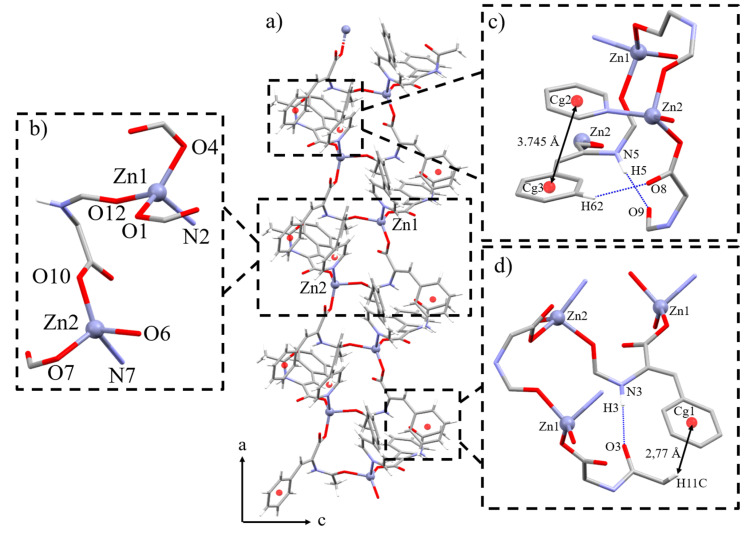
(**a**) General view of the polymeric chain of **1** along the *a* axis. In detail view of (**b**) *core* atoms of the polymeric chain. (**c**) N-H···O, C-H···O, and π·· π intramolecular interactions. (**d**) N-H···O and C-H···π intramolecular interactions. The phenyl, pyridyl, and other atoms which do not participate in any intramolecular interaction have been omitted for clarity in the detailed views.

Compound **1** crystallizes in the monoclinic P2_1_/c space group. It consists of a Zn(II) polymeric chain expanded along the *a* axis through two crystallographically different Zn(II) metal centers with slightly different bond lengths and bond angles. Both metal centers present a [ZnNO_3_] *core* exhibiting O-Zn-O bond angles between 101.6(1)° and 129.1(1)°, and O-Zn-N between 89.4(1)° and 139.1(2)° (Table 1; Figure 1a). The distortion on the tetrahedral or square planar geometry can be determined by the τ_4_ parameter [53], where the values range between 1.00 for a perfect tetrahedral geometry, to 0.00 for a perfect square planar geometry. In compound **1**, the two different Zn(II) metal centers present values of the τ_4_ parameter of 0.81 (Zn1) and 0.80 (Zn2), indicating similar distorted tetrahedral geometries.

The CP **1** is composed of dimeric units with their metal centers presenting the same coordination sphere, which contains two ACA and one 4-Phpy ligands each one. The ACA ligands display two types of coordination modes: monodentate *via* carboxylate group or bridge *via* carboxylate and carbonyl groups. Despite the two different roles of the ACA ligands in this compound, their carboxylate groups exhibit always a monodentate coordination mode (Figure 1b).

The Zn-O bond lengths are between 1.949(4) and 2.010(3) Å with no significant differences between the carboxylate and the carbonyl Zn-O bond lengths. In addition, the Zn-N bond lengths are slightly longer than the Zn-O bonds, with values of 2.054(4) and 2.072(4) Å. These values are similar to other Zn(II) compounds with Zn-O coordination through carbonyl groups [54,55,56] or carboxylate ligands which display monodentate coordination modes in combination with pyridine derivative ligands [57,58].

The intramolecular interactions of **1** are promoted by the bridging and monodentate ACA ligands. The bridging ligands act as hydrogen bond donors *via* NH and *m*-H moieties displaying three different interactions, two of them with oxygen atoms of carbonyl units (N-H···O), and one with an uncoordinated oxygen atom from a carboxylate group (C-H···O). Moreover, the bridging ACA ligands display a π···π interaction between its aromatic ring and the pyridyl ring of the 4-Phpy (Figure 1c). By contrast, the monodentate ACA ligands act as hydrogen bond donors by C-H···π interactions through their methyl groups with the phenyl unit of a bridging ACA ligand (Figure 1d; Table S1).

**Figure 2:**
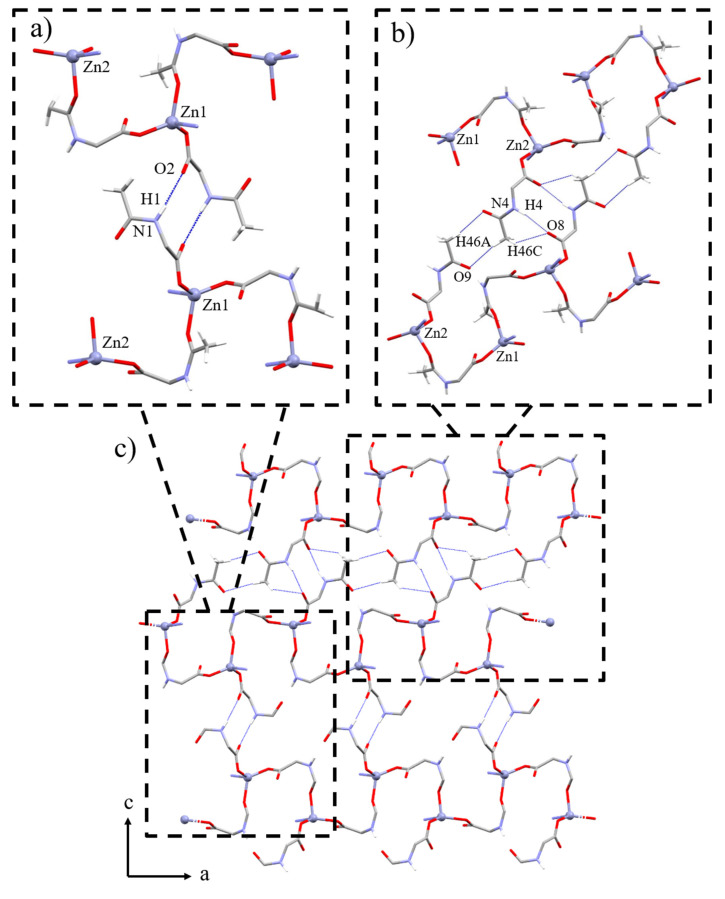
In detail view of: (**a**) N-H···O interactions. (**b**) N-H···O and C-H···O interactions through the polymeric chains. (**c**) General view of the 2D layers formed by intermolecular interactions along the *ac* plane in **1**. The phenyl, pyridyl and other atoms which do not participate in any of the mentioned interactions have been omitted for clarity.

Compound **1** expands its structure along the *c* direction through two groups of interactions in which only the monodentate ACA ligands participate. The former is composed of one strong H-bond between the amine unit and the uncoordinated oxygen atom from the carboxylate group (Figure 2a). The latter is formed of three moderate interactions [59], one H-bond between the same groups as the previous H-bond (N-H···O_COO_), and two C-H···O interactions, which assemble two methyl protons with one oxygen atom from a carbonyl and one from a monodentate carboxylate group of the same nearby polymeric chain (Figure 2b). These interactions expand the structure of **1** along the *c* direction, forming 2D layers through the *ac* plane in combination with the expansion of the polymeric chains (Figure 2c).

**Figure 3:**
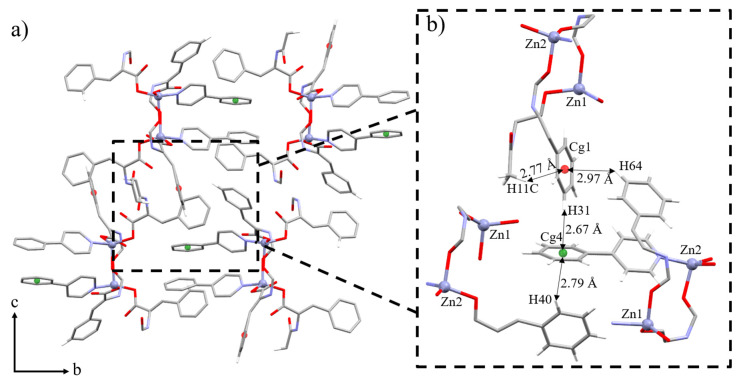
(**a**) General view of the supramolecular *bc* plane. (**b**) In-detail view of the former C-H···π interactions in **1**. The phenyl, pyridyl and other atoms which do not participate in any of the mentioned interactions have been omitted for clarity.

Furthermore, there are three C-H···π interactions, which expand the structure through the *bc* plane. In two of them the aromatic ring from a 4-Phpy connect two different polymeric chains throughout the *c* direction by a *m*-H and a *o*-H atoms of a bridged and a monodentate ACA ligands. In addition, the aromatic ring of the *m*-H atom participates in another C-H···π interaction with a bridged ACA ligand, expanding the structure through the *b* direction together with the intramolecular C-H···π interaction previously described (Table 1; Figure 3; Table S1). All this set of interactions expands the structure forming a 3D network.

### 2.3. Crystal Structures **2**·4CH_3_CN and **3**·4EtOH

**Figure 4:**
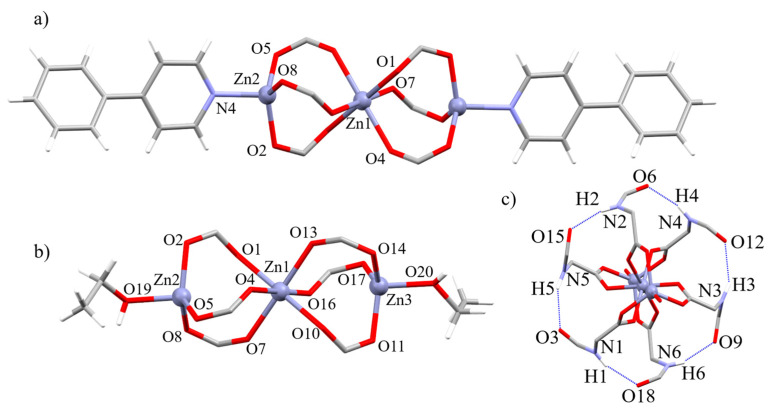
Molecular structure of complex: (**a**) **2**·4CH_3_CN and (**b**) **3**·4EtOH. (**c**) N-H···O intramolecular interactions of the ACA ligands in **2**·4CH_3_CN and **3**·4EtOH with labels assigned for complex **3**·4EtOH. The phenyl and other atoms which do not participate in any of the mentioned interactions have been omitted for clarity.

Compound **2**·4CH_3_CN crystallizes in the monoclinic P2_1_/c space group. On the other hand, compound **3**·4EtOH crystallizes in the monoclinic P2_1_ space group. Both compounds form linear trinuclear arrays presenting two different types of Zn(II) ions, one central and two terminal metal atoms, but in **2**·4CH_3_CN the terminal metal atoms are equal while in **3**·4EtOH they present slightly different bond lengths and angles. The central metal atoms for both compounds exhibited a [ZnO_6_] *core* formed by six carboxylate groups from six ACA ligands connected to the terminal metal atoms through six bridged coordination modes. The terminal metal atoms in **2**·4CH_3_CN present a [ZnO_3_N] *core* composed by three ACA ligands with bridged coordination modes and a 4-Phpy ligand which completes the coordination sphere (Figure 4a). Differently, in **3**·4EtOH the bridged ACA ligands are maintained but the 4-Phpy ligand is exchanged by an EtOH molecule, displaying a [ZnO_4_] *core* (Figure 4b). In addition, **2**·4CH_3_CN presents two pairs of co-crystallized CH_3_CN molecules while **3**·4EtOH presents four different co-crystallized EtOH molecules.

The distinction on the geometry of the hexacoordinated Zn(II) center between octahedral and trigonal prismatic is evaluated through the average twist angle (*ata*), but it must be noted that this value does not give information on the distortion of the geometry [60,61]. While higher *ata* around 60° belongs from octahedral geometry, the lower values close to 0° pertain to a trigonal prism geometry. Compound **2**·4CH_3_CN exhibits an *ata* value of 60.00° (torsion angles: 60.32°, 57.80°, 61.88°) and compound **3**·4EtOH presents an *ata* of 59.82° (torsion angles: 59.10°; 58.88°, 61.49°), both indicating an octahedral geometry with Zn-O bond lengths between 2.0861(15) and 2.1644(15) Å (**2**·4CH_3_CN) and between 2.150(3) and 2.172(3) Å (**3**·4EtOH), and bond angles ranging from 89.61(6) to 93.10(6)° in **2**·4CH_3_CN and between 87.06(9) to 92.96(9)° for **3**·4EtOH, indicating a slightly distortion on the geometry in both central metal atoms (Table 2 and Table 3).

Moreover, the four-coordinated centers present Zn-O bond lengths between 1.9254(15) and 1.9673(15) Å, a Zn-N bond length of 2.0358(17) Å and bond angles ranging between 99.97(7)° and 119.84(7)° in **2**·4CH_3_CN. Conversely, in compound **3**·4EtOH, the Zn-O bond lengths are between 1.934(2) and 1.985(2) Å and the bond angles range between 92.69(10)° and 123.27(10)°. The distortion of the four-coordinated geometry is evaluated using the τ_4_ parameter, presenting a value of 0.87 for **2**·4CH_3_CN and 0.87 and 0.86 for Zn2 and Zn3 respectively, in **3**·4EtOH. These values indicate a tetrahedral geometry with a slight distortion in all the four-coordinated metal atoms. Furthermore, in both compounds the values of bond lengths and angles are in accordance with similar structures consisting of linear trinuclear arrays bridged by carboxylate ligands with nitrogen atoms from pyridine derivative ligands [32,34], or oxygen atoms from solvent molecules, in the lateral positions [31,33,34].

Finally, both trinuclear structures display six moderate intramolecular N-H···O interactions between the amine and the carbonyl units of the amide moieties from the different bridged ACA ligands, connecting all the carboxylic ligands between them and supporting the disposition of the ligands in the molecular array. In **2**·4CH_3_CN, there are three different values for these interactions. Differently, in **3**·4EtOH, the structure shows six different values for the N-H···O interactions (Figure 4c; Tables S2 and S3).

### 2.4. Extended Structures of **2**·4CH_3_CN and **3**·4EtOH

**Figure 5:**
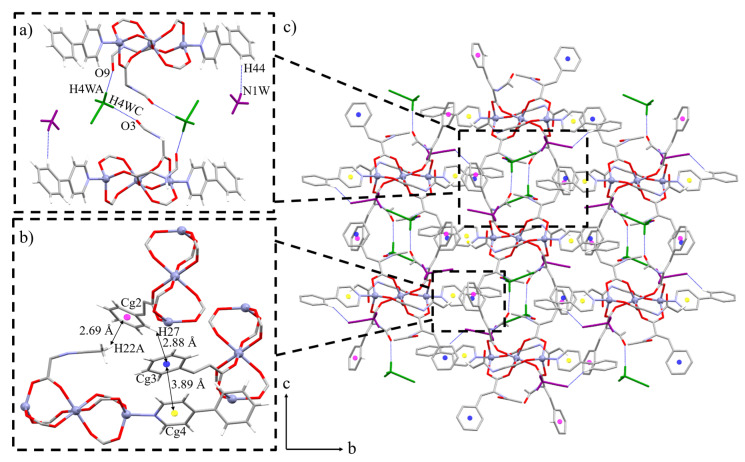
(**a**) In detail view of the C-H···N and C-H···O interactions of **2**·4CH_3_CN between the two types of co-crystallized acetonitrile molecules (highlighted in green and purple) and the trinuclear units which expand the structure along the *c* axis. (**b**) In detail view of the C-H···π and π··· π interactions which contribute to the formation of the *bc* plane. (**c**) General view of the resulting plane along the *bc* axes due to the C-H···O and the planar interactions. The phenyl, pyridyl, and other atoms which do not participate in any of the mentioned interactions have been omitted for clarity.

The intermolecular interactions of **2**·4CH_3_CN are mainly through C-H···π associations between the ACA ligands and C-H···O/C-H···N interactions between the ACA ligands and the two co-crystallized CH_3_CN molecules, which present different behaviors. One co-crystallized CH_3_CN molecule exhibits a moderate C-H···N interaction without propagation of the structure. Otherwise, the second CH_3_CN molecule interacts through two protons of its -CH_3_ moiety with two carbonyl oxygen atoms from nearby trinuclear units, forming two strong C-H···O interactions which expand the structure through the *c* axis (Figure 5a).

Furthermore, three ACA ligands of three different trinuclear units show two C-H···π interactions. In the first interaction, the H-donor is a -CH_3_ moiety from a first trinuclear unit, which interacts with an aromatic ring from a nearby ACA ligand (Figure 5b, pink centroid). The second interaction associates the *o*-H atom of the previous aromatic ACA ring with a phenyl ring from a neighboring ACA ligand (Figure 5b, blue centroid). In addition, the same phenyl ring which displays the second C-H···π interaction also promotes a π···π stacking with a pyridyl ring from a 4-Phpy of the first trinuclear unit (Figure 5b, blue and yellow centroids). This set of interactions together with the C-H···O interactions previously mentioned, expand the structure through the *bc* axes, forming a 2D plane (Table 2; Figure 5c).

On the other hand, in **3**·4EtOH, the co-crystallized and coordinated EtOH molecules as well as two of the ACA aromatic rings from the trinuclear units are involved in most of the interactions. The co-crystallized EtOH molecules connect each trinuclear unit to two neighboring ones through two slightly different patterns of intermolecular interactions, while the C-H···π interactions join each trinuclear unit to another four.

**Figure 6:**
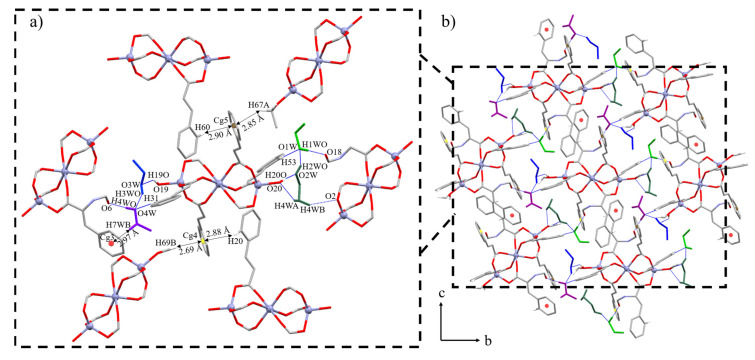
(**a**) In detail view of the H-bonds, C-H···O and planar interactions of complex **3**·4EtOH. (**b**) General view of the supramolecular 2D plane formed along the *bc* axes. The different types of co-crystallized EtOH molecules have been highlighted in different colors and the phenyl, pyridyl, and other atoms which do not participate in any of the mentioned interactions have been omitted for clarity.

Both patterns of the co-crystallized EtOH molecules start with an H-bond between the hydroxyl groups of a coordinated and a co-crystallized EtOH molecules, with the difference that this H-bond presents a moderate strength for one co-crystallized EtOH molecule (Figure 6a, dark blue EtOH molecule), while for the other present a strong strength (Figure 6a, dark green EtOH molecule). Moreover, each of these co-crystallized EtOH molecules are held together with another co-crystallized EtOH molecule *via* a moderate H-bond between their hydroxyl groups. The patterns are completed by a strong H-bond between the hydroxyl groups of the second co-crystallized EtOH molecules and the oxygen atoms from carbonyl groups of neighboring trinuclear units. These interactions are also supported by a strong C-H···O interaction between the *o*-H of ACA ligands from the central trinuclear unit and the hydroxyl groups from these second co-crystallized EtOH molecules, which also connect the trinuclear units directly through the second co-crystallized EtOH molecules (Table 3; Figure 6a, light green and purple EtOH molecules).

Moreover, two of the co-crystallized EtOH molecules exhibit other weak interactions with the same trinuclear units connected by the previously mentioned interactions. The first interacts with a hydroxyl group from a coordinated EtOH molecule through a moderate C-H···O interaction with its methyl moiety, which also display another moderate C-H···O interaction with an oxygen atom from a carbonyl group of a second trinuclear unit (Figure 6a, dark green EtOH molecule). The second shows a C-H···π interaction between a proton from its -CH_2_ group with an aromatic ring from an ACA ligand (Figure 6a, purple and red centroids).

Finally, two ACA ligands from each trinuclear unit displays four C-H···π interactions. Two of them are formed between *o*-H atoms from two different nearby trinuclear units with two different aromatic rings from ACA ligands of the same central trinuclear unit. The remaining two C-H···π interactions associate two hydrogen atoms from two -CH_2_ moieties of coordinated EtOH molecules from different trinuclear units with the same aromatic rings as the previous planar interactions (Figure 6a, brown and yellow centroids). All this set of interactions expands the structure of **3**·4EtOH along the *bc* axes, forming a 2D plane (Figure 6b).

### 2.5. Hirshfeld Surface Analysis

Hirshfeld surface analysis of complexes **2**·4CH_3_CN and **3**·4EtOH have been performed with CrystalExplorer17.5 [62]. All the surfaces have been calculated at an isovalue of 0.5 e·au^−3^. In addition, the disorder present in a co-crystallized EtOH molecule of **3**·4EtOH has been identified, and the fractional occupancy has been corrected to 1 in the .cif file. Moreover, the Electrostatic Surface Potential (ESP) of **2**·4CH_3_CN and **3**·4EtOH have been calculated by Density Functional Theory (DFT) at the B3LYP/6-31 G(d,p) level using TONTO [63]. Both ESP surfaces have been represented over the range −0.03 au (red), through zero (white), to 0.03 au (blue). Unfortunately, the Hirshfeld Surface Analysis of the CP **1** as well as their ESP calculations could not be performed owing to its polymeric array.

Hirshfeld surfaces, in combination with 2D fingerprint plots, are a powerful graphical tool to evaluate supramolecular interactions present in crystal structures. The surface mapping facilitates their identification while the fingerprint plot outlines the distances between the atoms involved in these contacts. Furthermore, the ESP calculations allow the identification of electropositive (blue) and electronegative (red) regions, whose complementarity supports the presence of supramolecular interactions [64].

**Figure 7:**
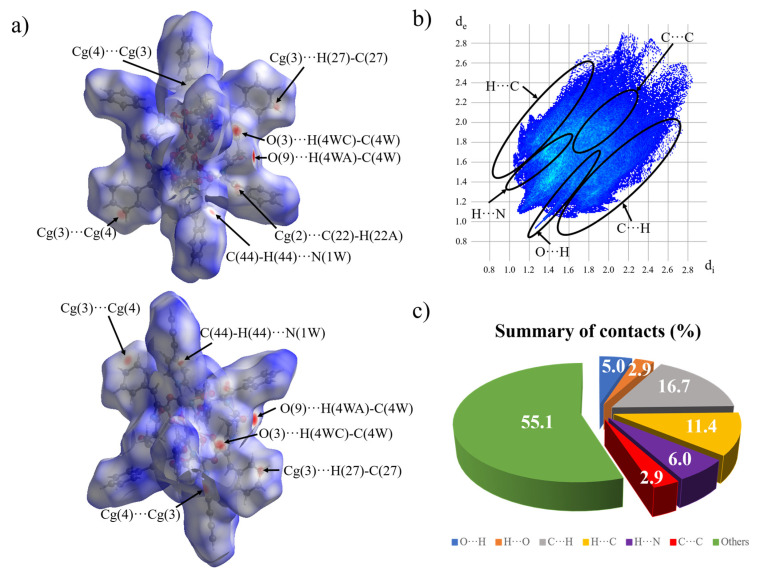
(**a**) Hirshfeld surface d_norm_ representations, (**b**) 2D fingerprint plot highlighting the intermolecular interactions, and (**c**) graphical summary of the percentage of each contact involved in the Hirshfeld surface of **2**·4CH_3_CN.

**Figure 8:**
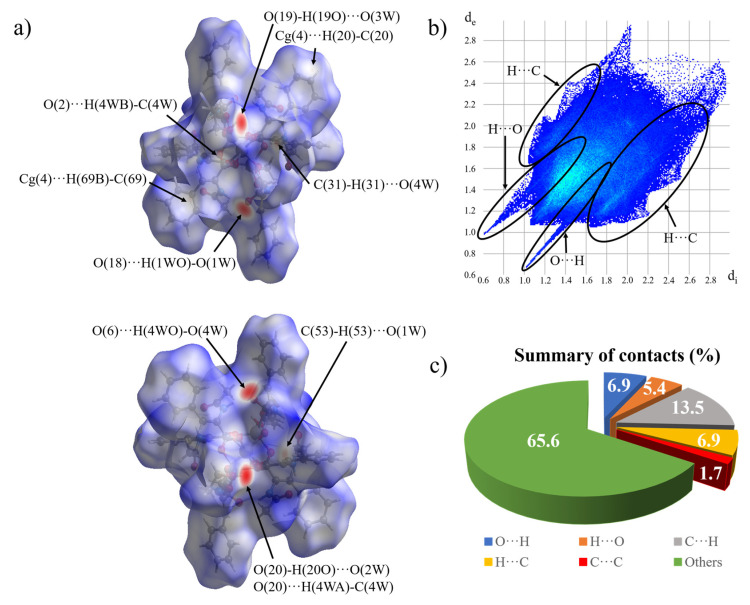
(**a**) Hirshfeld surface d_norm_ representations, (**b**) 2D fingerprint plot highlighting the intermolecular interactions, and (**c**) graphical summary of the percentage of each contact involved in the Hirshfeld surface of **3**·4EtOH.

The main associations of **2**·4CH_3_CN and **3**·4EtOH are highlighted in the Hirshfeld surface views mapped with d_norm_ in Figure 7 and Figure 8. Additional Hirshfeld surface mappings, ESP representations and 2D fingerprint plots of the co-crystallized solvent molecules of **2**·4CH_3_CN and **3**·4EtOH are illustrated in the Figures S11 and S12.

In the d_norm_ representations of the two trinuclear complexes, the different intermolecular H-bonds, C-H···O/N and planar interactions can be identified as red regions supporting the interactions previously described in the corresponding structural sections (Figure 7a and Figure 8a). The 2D fingerprint plot of **3**·4EtOH shows the presence of prominent O···H/H···O contacts represented as sharp spikes in the d_e_ + d_i_ ≈ 1.6–1.8 Å range. Differently, in complex **2**·4CH_3_CN these regions seem asymmetric and show more discrete spikes (Figure 7b and Figure 8b). The reason of this asymmetric fingerprint plot is the different type of interactions of the co-crystallized acetonitrile molecules with the trinuclear units of **2**·4CH_3_CN. Whereas one type of co-crystallized acetonitrile molecules act as H-bond acceptor through its nitrogen atom (C-H···N interaction), the other acetonitrile molecule interact with the carbonyl oxygen atoms of the trinuclear units through two types of C-H···O interactions, displaying intermolecular interactions with different H-acceptors where the trinuclear unit act as H-bond donor for the C-H···N and H-bond acceptor for the C-H···O interactions. These interactions present different distances (d_e_ + d_i_ ≈ 2.4–2.6 Å for the H···N contacts and d_e_ + d_i_ ≈ 2.1–2.3 Å for the O···H) and this is illustrated in the 2D fingerprint plot with asymmetric spikes.

Moreover, if we compare the summary of contacts of the two trinuclear complexes, the H···O/O···H contacts present a 12.3% of the surface associated in **3**·4EtOH, which means a 4.4% of difference respect to **2**·4CH_3_CN, with a 7.9% of the surface implied (Figure 7c and Figure 8c). This difference could be justified owing to the higher number of H···O/O···H contacts observed in **3**·4EtOH (eight interactions) in comparison with **2**·4CH_3_CN (four interactions), as well as the shorter H···A distances observed in the 2D fingerprint plot of **3**·4EtOH respect to the plot of **2**·4CH_3_CN. Furthermore, the presence of H···N contacts in **2**·4CH_3_CN also stands out, with a 6.0% of the surface implied, while in **3**·4EtOH there is not implied surface in the H···N contacts (Table 4).

The planar interactions present in the two trinuclear compounds have been confirmed using curvedness mapping of the involved regions in these interactions. In addition, ESP surfaces have been used to check the charge complementarity between the parts which participate in these associations (Figures S13 and S14). The general fingerprint plots of Figure 7b and Figure 8b also display a “wings” shape indicating the presence of C-H···π interactions [65], which in combination with the summary of contacts of each compound show a difference in the percentage of the surface involved in the d_norm_ Hirshfeld surface of **2**·4CH_3_CN compared with **3**·4EtOH. Compound **2**·4CH_3_CN presents a percentage of C···H/H···C contacts with a 28.1% of the surface implied (four interactions), which is a 7.7% of difference compared with **3**·4EtOH (five interactions). This difference could be explained owing to the presence of the 4-Phpy ligand in **2**·4CH_3_CN, which apart from participating in planar interactions, also perform C···H and H···C weak contacts, which cannot be considered as planar interactions but increase the percentage of the surface implied in the Hirshfeld mappings and could have some influence in the crystal packing through cooperative effects (Table 4). Differently, in **3**·4EtOH, the exchange of the 4-Phpy molecules by coordinated EtOH molecules displays a decrease of the C···H/H···C contacts, even though the number of C-H···π interactions (five interactions) is higher in **3**·4EtOH than in **2**·4CH_3_CN (Table 4). Moreover, the ESP surfaces of **2**·4CH_3_CN and **3**·4EtOH show the charge complementarity between electronegative charged aromatic rings from the ACA ligands and electropositive charged hydrogen atoms from the ACA and 4-Phpy aromatic rings and the CH_3_ moieties, involved in all the planar interactions of the two compounds.

Additionally, the π···π interactions present in **2**·4CH_3_CN are also observed in the summary of contacts involved in the d_norm_ representation and, although their identification in the general 2D fingerprint plots is difficult to distinguish, the ESP surface show well the charge complementarity between the electronegative charged ACA rings and the electropositive 4-Phpy rings. Moreover, the summary of contacts shows a higher percentage of the surface implied in **2**·4CH_3_CN (2.9%) compared with **3**·4EtOH (1.7%) for the C···C contacts, which is probably done to the presence of π···π interactions in **2**·4CH_3_CN.

**Figure 9:**
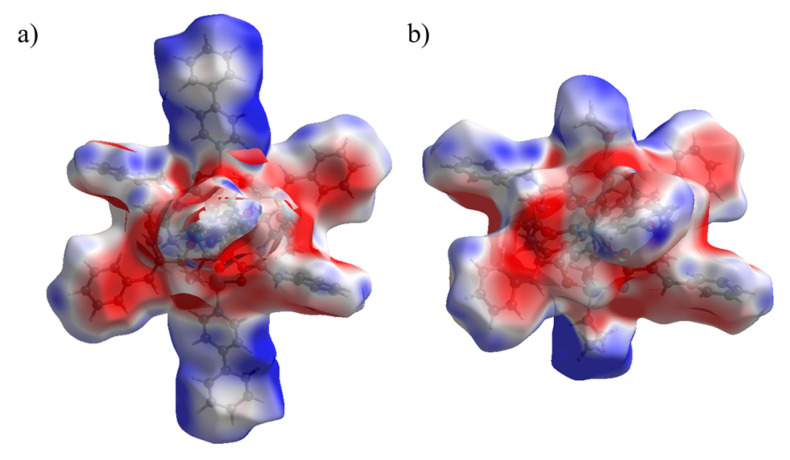
Electrostatic Surface Potential (ESP) surfaces highlighting the electropositive (blue) and electronegative (red) regions over the range ±0.03 au for complexes: (**a**) **2**·4CH_3_CN and (**b**) **3**·4EtOH.

It is noteworthy that in the three structures the ACA aromatic rings are placed avoiding the planar stacking between them and favoring the formation of C-H···π interactions, while the π···π interactions are formed between aromatic rings of ACA (electron donors) and 4-Phpy (electron acceptors) in **1** (intramolecular interaction) and **2**·4CH_3_CN (intermolecular interaction). The ESP surfaces reveal that the lack of charge complementarity between ACA rings could be the factor which promotes the formation of C-H···π interactions, whereas the π···π stackings are formed by mixed ACA and 4-Phpy rings, whose charge complementarity has been observed by the ESP surfaces (Figure 9). This effect has also been observed in recently synthesized Zn(II) complexes containing ACA and 4-Phpy ligands by our group [42]. The reason of the electronegative charge in the ACA aromatic ring could be the combination of their double bond and the acetamide moiety, which by resonance effect, confers to the aromatic ring an electronegative charge, while for the 4-Phpy the nitrogen atom, which is more electronegative than the rest of the carbon atoms of the rings, pull up the charge from the pyridyl and, to a lesser extent, from phenyl ring, providing an electropositive charge to these rings.

## 3. Conclusions

In this paper, we present the reaction of Zn(OAc)_2_·2H_2_O in combination with HACA and 4-Phpy in EtOH as solvent, yielding the 1D CP **1** with tetrahedral geometry, which extends its structure through a carboxylate-carbonyl bridge of their ACA ligands. Its recrystallization in CH_3_CN and EtOH yields two trinuclear complexes with pinwheel arrays and formula [Zn_3_(µ-ACA)_6_(L)_2_] (L= 4-Phpy (**2**), EtOH (**3**)). Moreover, compound **2** has been obtained by an alternative method reacting Zn(OAc)_2_·2H_2_O, HACA, and 4-Phpy in a 1:2:2 ratio using CH_3_CN as solvent at RT. These trinuclear complexes contain two types of Zn(II) metal centers, one with octahedral (central) and the other two with tetrahedral geometries.

The crystal structure of the three compounds have been elucidated, and their molecular and supramolecular interactions have been discussed obtaining 2D arrays for the two trinuclear complexes and a 3D network for the CP **1**. Furthermore, Hirshfeld surface analysis has been used to analyze and compare the supramolecular interactions of the trinuclear complexes. The Hirshfeld surfaces as well as the ESP mappings allow us the identification of different type of supramolecular interactions supporting the information obtained by the structural study. Interestingly, the ESP calculations show that the aromatic rings of the 4-Phpy act as electron acceptors and the ACA aromatic rings act as electron donors and thus, favoring the formation of π···π interactions between 4-Phpy and ACA aromatic rings (**1** and **2**·4CH_3_CN). Otherwise, the lack of charge complementarity between ACA aromatic rings lead to the formation of C-H···π interactions between nearby ACA ligands in the three structural arrays. Finally, the co-crystallized solvent molecules in structures **2**·4CH_3_CN and **3**·4EtOH also have an important contribution in the crystal packing, connecting the trinuclear arrays by different types of interactions, which have also been identified.

## 4. Experimental Section

### 4.1. Materials and Methods

Zinc(II) acetate dihydrate (Zn(OAc)_2_·2H_2_O), α-acetamidocinnamic acid (HACA), 4-phenylpyridine (4-Phpy), ethanol (EtOH), and acetonitrile (CH_3_CN) as solvents, were purchased from Sigma-Aldrich (Darmstadt, Germany). Deuterated dimethylsulfoxide (dmso-*d*_6_) was used for the NMR experiments and was purchased from Eurisotop (Saint-Aubin, France). All of them were used without further purification. All the reactions and manipulations were carried out in air at room temperature (RT). Elemental analyses (C, H, and N) were carried on a Thermo Scientific (Waltham, MA, USA) Flash 2000 CHNS Analyzer. HR-ESI-MS measurements were recorded after dissolving the corresponding solid of CP **1** in CH_3_CN and EtOH in a MicroTOF-Q (Bruker Daltonics GmbH, Bremen, Germany) instrument equipped with an electrospray ionization source (ESI) in positive mode. Na^+^ ions come from the EtOH solvent which contains < 50 ppb. Conditions were those used in routine experiments. The nebulizer pressure was 1.5 bar, the desolvation temperature was 180 °C, dry gas at 6 L min^−1^, the capillary counter-electrode voltage was 5 kV, and the quadrupole ion energy, 5.0 eV. FTIR-ATR spectra were recorded on a Perkin Elmer (Waltham, MA, USA) spectrometer, equipped with an attenuated total reflectance (ATR) accessory model MKII Golden Gate with a diamond window in the range 4000–500 cm^−1^. ^1^H, ^13^C{^1^H}, and DEPT-135 NMR spectra were recorded on an NMR-FT Bruker 360 and 400 (Karlsruhe, Germany) MHz spectrometers in dmso-*d*_6_ solution at RT. All chemical shifts (δ) are given in ppm relative to tetramethylsilane (TMS) as internal standard.

### 4.2. Synthesis of [Zn_2_(µ-O,O’-ACA)_2_(ACA)_2_(4-Phpy)_2_]_n_ (**1**)

An EtOH solution (20 mL) of Zn(OAc)_2_·2H_2_O (152 mg, 0.692 mmol) was added dropwise to a solution of HACA (283 mg, 1.38 mmol) and 4-Phpy (215 mg, 1.39 mmol) in EtOH (10 mL) as solvent. The resulting solution was stirred for 18 h at RT until a white solid precipitated. The white precipitate was washed with 10 mL of cold EtOH, filtered and dried under vacuum. Suitable colorless crystals were obtained by keeping the mother liquors on the fridge for 5 days. Yield: 382 mg (88%) (based on Zn).

Elemental analysis calc.(%) for C_66_H_58_Zn_2_N_6_O_12_ (1257.98): C 63.01, H 4.65, N 6.68; found: C 63.10, H 4.49, N 6.67. HR-MS (ESI^+^, CH_3_CN): *m*/*z* (%) = 495.0505 (100%) (calc. for [Zn(ACA)_2_ + Na]^+^ = 495.0505); 650.1235 (100%) (calc. for [Zn(ACA)_2_(4-Phpy) + Na]^+^ = 650.1240); 967.1127 (89%) (calc. for [Zn_2_(ACA)_4_ + Na]^+^ = 967.1118). HR-MS (ESI+, EtOH): m/z (%) = 495.0505 (100%) (calc. for [Zn(ACA)_2_ + Na]^+^ = 495.0505). FTIR-ATR (wavenumber, cm^−1^): 3217(w) [ν(N-H)], 3161(w) [ν(N-H)], 3054(m) [ν(C-H)_ar_], 2985(w) [ν(C-H)_alk_], 2859(w) [ν(C-H)_al_], 1672(w) [ν(C=O)], 1651(w), 1583(s) [ν_as_(COO)], 1551(sh) [ν(C=C/C=N)], 1509(m), 1489(m), 1446(w), 1422(w), 1379(s) [ν_s_(COO)], 1346(s) [δ(C=C/C=N)], 1311(m), 1279(m), 1226(w), 1210(w), 1184(w), 1158(w), 1141(w), 1126(w), 1072(w) [δ_ip_(C-H)], 1046(w), 1030(w), 1012(w), 1002(m) [δ_ip_(C-H)], 971(w), 931(w), 838(w), 794(w), 765(s) [δ_oop_(C-H)], 733(m), 688(s) [δ_oop_(C-H)], 622(m), 591(m). ^1^H NMR (360 MHz; dmso-*d*_6_; Me_4_Si; 298 K): δ = 9.30 [2H, s, N*H*_ACA_], 8.77 [2H, d. ^3^J = 5.8 Hz, *o*-*H*_py_,_4-Phpy_], 7.91 [2H, d, ^3^J = 5.8 Hz, *m*-*H*_py_,_4-Phpy_], 7.86 [2H, d, ^3^J = 7.6 Hz, *o*-*H*_ph_,_4-Phpy_], 7.55 [7H, m, *o*-*H*_ACA_ + *m*-*H*_ph_,_4-Phpy_ + *p*-*H*_ph_,_4-Phpy_], 7.36 [4H, t, ^3^J = 7.5 Hz, *m*-*H*_ACA_], 7.30 [2H, s, NH-C-C*H*_ACA_], 7.27 [2H, d, ^3^J = 7.5 Hz, *p*-*H*_ACA_], 1.99 [6H, s, CO-C*H*_3_,_ACA_]. ^13^C{^1^H} NMR (400 MHz; dmso-*d*_6_; Me_4_Si; 298 K): δ = 170.8 [NH-*C*O_ACA_], 168.5 [*C*O_2_,_ACA_], 150.0 [*o*-*C*_py_,_4-Phpy_], 149.1 [Ph-*C*_py_,_4-Phpy_], 136.4 [Py-*C*_ph_,_4-Phpy_], 135.1 [O_2_C-*C*_ACA_], 130.0 [*o*-*C*_ACA_], 129.8 [HN-C-CH-*C*_ACA_], 129.4 [*m*-*C*_ph_,_4-Phpy_ + *p*-*C*_ph_,_4-Phpy_], 129.1 [*p*-*C*_ACA_], 128.4 [*m*-*C*_ACA_], 128.2 [NH-C-*C*H_ACA_], 127.2 [*o*-*C*_ph_,_4-Phpy_], 122.1 [*m*-*C*_py_,_4-Phpy_], 23.1 [CO-*C*H_3_,_ACA_]. DEPT-135 NMR (360 MHz; dmso-*d*_6_; Me_4_Si, 298 K): δ = 149.9 [*o*-*C*_py_,_4-Phpy_], 130.0 [*o-C*_ACA_], 129.4 [*m*-*C*_ph_,_4-Phpy_ + *p*-*C*_ph_,_4-Phpy_], 129.1 [*p*-*C*_ACA_], 128.4 [*m*-*C*_ACA_], 128.2 [O_2_C-C-*C*_ACA_], 127.2 [*o*-*C*_ph_,_4-Phpy_], 122.1 [*m*-*C*_py_,_4-Phpy_], 23.1 [CO-*C*H_3_,_ACA_].

### 4.3. Synthesis of [Zn_3_(µ-ACA)_6_(4-Phpy)_2_] (**2**)

*Method A*. A small amount of **1** (56.5 mg, 0.0449 mmol) was dissolved in CH_3_CN (50 mL) using a water bath at 60 °C. The solution was heated and stirred for 4 h until **1** is dissolved. The clear solution was stirred for 3 h at RT and then was kept on the fridge for 14 h. The solution was evaporated under vacuum until a white crystalline powder precipitated. The powder was washed with 10 mL of cold CH_3_CN, filtered and dried under vacuum. Suitable colorless crystals of [Zn_3_(µ-ACA)_6_(4-Phpy)_2_]·4CH_3_CN (**2**·4CH_3_CN) were obtained by recrystallization of **1** in CH_3_CN and slow evaporation of the solution at RT for 8 days. Yield: 37.8 mg (77%) (based on Zn).

*Method B*. A hot CH_3_CN solution (30 mL, 60 °C) of Zn(OAc)_2_·2H_2_O (150 mg, 0.683 mmol) was added dropwise to a solution of HACA (280 mg, 1.36 mmol) and 4-Phpy (217 mg, 1.40 mmol) in CH_3_CN (20 mL) as solvent at RT. After two minutes, a white solid precipitated (**2**) and the dispersion was stirred for 18 h. The white precipitate was washed with 10 mL of cold CH_3_CN, filtered and dried under vacuum. Suitable colorless crystals of [Zn_3_(µ-ACA)_6_(4-Phpy)_2_]·4CH_3_CN (**2**·4CH_3_CN) were obtained by recrystallization of **2** in CH_3_CN and slow evaporation of the solution at RT for 6 days. Yield: 298 mg (76%) (based on Zn).

Elemental analysis calc.(%) for C_88_H_78_Zn_3_N_8_O_18_ (1731.77): C 61.03, H 4.54, N 6.47; found: C 60.77, H 4.46, N 6.32. FTIR-ATR (wavenumber, cm^−1^): 3386(w), 3236(m) [ν(N-H)], 3054–3021(br) [ν(C-H)_ar_], 2980–2929(br) [ν(C-H)_alk_ + ν(C-H)_al_], 1678(m) [ν(C=O)], 1653(sh), 1616(w), 1573(s) [ν_as_(COO)], 1536(m) [ν(C=C/C=N)], 1505(m), 1488(m), 1446(w), 1390(s) [ν_s_(COO)], 1366(s), 1347(s) [δ(C=C/C=N)], 1270(m), 1258(sh), 1228(w), 1212(m), 1182(w), 1157(w), 1122(w), 1075(w) [δ_ip_(C-H)], 1030(w) [δ_ip_(C-H)], 1013(w) [δ_ip_(C-H)], 959(w), 925(w), 897(w), 838(w), 791(m), 760(s) [δ_oop_(C-H)], 746(sh), 688(s) [δ_oop_(C-H)], 624(m), 616(sh), 590(s), 579(s), 561(m). ^1^H NMR (400 MHz; dmso-*d*_6_; Me_4_Si; 298 K): δ = 9.19 [6H, s, N*H*_ACA_], 8.66 [4H, d. ^3^J = 6.2 Hz, *o*-*H*_py_,_4-Phpy_], 7.82 [4H, d, ^3^J = 7.3 Hz, *o*-*H*_ph_,_4-Phpy_], 7.76 [4H, d, ^3^J = 6.2 Hz, *m*-*H*_py_,_4-Phpy_], 7.53 [18H, m, *o*-*H*_ACA_ + *m*-*H*_ph_,_4-Phpy_ + *p*-*H*_ph_,_4-Phpy_], 7.35 [12H, t, ^3^J = 7.7 Hz, *m*-*H*_ACA_], 7.29 [6H, d, ^3^J = 7.3 Hz, *p*-*H*_ACA_], 7.25 [6H, s, NH-C-C*H*_ACA_], 1.96 [18H, s, CO-C*H*_3_,_ACA_]. ^13^C{^1^H} NMR (360 MHz; dmso-*d*_6_; Me_4_Si; 298 K): δ = 170.7 [NH-*C*O_ACA_], 168.5 [*C*O_2_,_ACA_], 150.0 [*o*-*C*_py_,_4-Phpy_], 148.8 [Ph-*C*_py_,_4-Phpy_], 136.6 [Py-*C*_ph_,_4-Phpy_], 135.1 [O_2_C-*C*_ACA_], 129.9 [*o*-*C*_ACA_], 129.7 [HN-C-CH-*C*_ACA_], 129.4 [*m*-*C*_ph_,_4-Phpy_+ *p*-*C*_ph_,_4-Phpy_], 129.1 [*p*-*C*_ACA_], 128.4 [*m*-*C*_ACA_], 128.2 [NH-C-*C*H_ACA_], 127.2 [*o*-*C*_ph_,_4-Phpy_], 121.9 [*m*-*C*_py_,_4-Phpy_], 23.1 [CO-*C*H_3_,_ACA_]. DEPT-135 NMR (360 MHz; dmso-*d*_6_; Me_4_Si, 298 K): δ = 150.0 [*o*-*C*_py_,_4-Phpy_], 129.9 [*o*-*C*_ACA_], 129.4 [*m*-*C*_ph_,_4-Phpy_+ *p*-*C*_ph_,_4-Phpy_], 129.1 [*p*-*C*_ACA_], 128.4 [*m*-*C*_ACA_], 128.2 [O_2_C-C-*C*_ACA_], 127.2 [*o*-*C*_ph_,_4-Phpy_], 122.0 [*m*-*C*_py_,_4-Phpy_], 23.1 [CO-*C*H_3_,_ACA_].

### 4.4. Synthesis of [Zn_3_(µ-ACA)_6_(EtOH)_2_] (**3**)

A small amount of **1** (57.1 mg, 0.0454 mmol) was dissolved in EtOH (40 mL). The solution was stirred for 2 h until **1** is dissolved. The clear solution was stirred for 17 h at RT and then evaporated under vacuum and kept on the fridge for 3 days. After this time, a small amount of a white crystalline powder (**3**) was obtained. The powder was washed with 10 mL of cold EtOH, filtered and dried under vacuum. Suitable colorless crystals of [Zn_3_(µ-ACA)_6_(EtOH)_2_]·4EtOH (**3·**4EtOH) were obtained by recrystallization of **1** and slow evaporation of the solution at RT for 2 days. Yield: 18.9 mg (46%) (based on Zn).

Elemental analysis calc.(%) for C_70_H_72_Zn_3_N_6_O_20_ (1513.52): C 55.55, H 4.79, N 5.55; found: C 55.37, H 4.64, N 5.42. FTIR-ATR (wavenumber, cm^−1^): 3308–3275(w) [ν(O-H)EtOH], 3227(w) [ν(N-H)], 3166(w), 3055–3026(w) [ν(C-H)_ar_], 2976–2881(w) [ν(C-H)_alk_ + ν(C-H)_al_], 1674(w), 1652(w) [ν(C=O)], 1585(sh), 1563(s) [ν_as_(COO)], 1539(s) [ν(C=C/C=N)], 1516(sh), 1492(m), 1446(m), 1392(s) [ν_s_(COO)], 1345(s) [δ(C=C/C=N)], 1274(m), 1212(w), 1183(w), 1119(w), 1091(w), 1080(w), 1047(m) [δ_ip_(C-H)], 1013(w) [δ_ip_(C-H)], 962(w) [δ_ip_(C-H)], 926(w), 878(w), 846(w), 790(m), 764(m) [δ_oop_(C-H)], 744(m) [δ_oop_(C-H)], 689(s) [δ_oop_(C-H)], 591(s), 580(sh). ^1^H NMR (360 MHz; dmso-*d*_6_; Me_4_Si; 298 K): δ = 9.20 [6H, s, N*H*_ACA_], 7.51 [12H, d, ^3^J = 6.9 Hz, *o*-*H*_ACA_], 7.35 [12H, t, ^3^J = 7.1 Hz, *m*-*H*_ACA_], 7.29 [6H, d, ^3^J = 6.8 Hz, *p*-*H*_ACA_], 7.24 [6H, s, NH-C-C*H*_ACA_], 4.39 [2H, t, ^3^J = 4.9 Hz, O*H*,_EtOH_], 3.45 [4H, m, C*H*_2_,_EtOH_], 1.95 [18H, s, CO-C*H*_3_,_ACA_], 1.05 [6H, t, ^3^J = 7.0 Hz, C*H*_3_,_EtOH_]. ^13^C{^1^H} NMR (360 MHz; dmso-*d*_6_; Me_4_Si; 298 K): δ = 170.6 [NH-*C*O_ACA_], 168.5 [*C*O_2_,_ACA_], 135.1 [O_2_C-*C*_ACA_], 129.7 [HN-C-CH-*C*_ACA_], 129.4 [*o*-*C*_ACA_], 129.2 [*p*-*C*_ACA_], 128.4 [*m*-*C*_ACA_], 128.3 [NH-C-*C*H_ACA_], 56.1 [*C*H_2_,_EtOH_], 23.1 [CO-*C*H_3_,_ACA_], 18.6 [*C*H_3_,_EtOH_]. DEPT-135 NMR (360 MHz; dmso-*d*_6_; Me_4_Si; 298 K): δ = 129.4 [*o*-*C*_ACA_], 129.2 [*p*-*C*_ACA_], 128.4 [*m*-*C*_ACA_], 128.3 [NH-C-*C*H_ACA_], 56.1 [*C*H_2_,_EtOH_], 23.1 [CO-*C*H_3_,_ACA_], 18.6 [*C*H_3_,_EtOH_].

## 5. X-ray Crystallographic Data

For compounds **1**, **2**·4CH_3_CN, and **3**·4EtOH, a colorless prism-like specimens were used for the X-ray crystallographic analysis. The X-ray intensity data were measured on a D8 Venture system equipped with a multilayer monochromate and a Mo microfocus (λ = 0.71073 Å). For **1**, **2**·4CH_3_CN, and **3**·4EtOH, the frames were integrated with the Bruker SAINT Software package using a narrow-frame algorithm. All hydrogen atoms were refined using a riding model (AFIX) with an isotropic temperature factor equal to 1.2, the equivalent temperature factor of the atom to which are linked and thus, the bond lengths of X-H were fixed. The structures were solved and refined using SHELX (version 2018/3) [66]. For **1**, the integration of the data using a monoclinic unit cell yielded a total of 80,518 reflections to a maxim θ angle of 30.70° (0.70 Å), of which 18,035 were independent (average redundancy 4.465, completeness = 98.4%), R_int_ = 20.23%, R_sig_ = 19.69%) and 8129 (45.07%) were greater than 2σ(F^2^). The calculated minimum and maximum transmission coefficients (based on crystal size) are 0.6289 and 0.7461. For **2**·4CH_3_CN, the integration of the data using a monoclinic unit cell yielded a total of 141282 reflections to a maxim θ angle of 30.55° (0.70 Å), of which 13913 were independent (average redundancy 10.155, completeness = 99.8%), R_int_ = 6.04%, R_sig_ = 3.35%) and 10988 (78.98%) were greater than 2σ(F^2^). The calculated minimum and maximum transmission coefficients (based on crystal size) are 0.6411 and 0.7461. For **3**·4EtOH, the integration of the data using a monoclinic unit cell yielded a total of 25525 reflections to a maxim θ angle of 30.97° (0.69 Å), of which 25,525 were independent (average redundancy 1.000, completeness = 99.7%), R_int_ = 4.21%, R_sig_ = 5.58%) and 20515 (80.37%) were greater than 2σ(F^2^). The calculated minimum and maximum transmission coefficients (based on crystal size) are 0.6349 and 0.7461.

For **1**, **2**·4CH_3_CN and **3**·4EtOH, the final cell constants and volume are based upon the refinement of the XYZ-centroids of reflections above 2θ σ(I). Data were corrected for absorption effects using the Multi-Scan method (SADABS). Crystal data and relevant details of structure refinement for compounds **1**, **2**·4CH_3_CN and **3**·4EtOH, are reported in Table 5. CCDC 2016324 (**1**), 2016325 (**2**·4CH_3_CN), and 2016323 (**3**·4EtOH) contain the Supplementary Information of this paper. Molecular graphics were generated using Mercury 4.3.1 software [67,68] with POV-Ray Package [69]. Color codes for all molecular graphics: dark blue (Zn), red (O), light blue (N), dark gray (C), and white (H).

## Figures and Tables

**Table 1:** Selected bond lengths (Å), bond angles (°), and intermolecular interactions (Å) of compound **1**.

Bond Lengths (Å)					
Zn(1)-O(1)	1.949(4)		Zn(2)-O(7)		1.979(3)
Zn(1)-O(4)	1.977(3)		Zn(2)-O(10)#1		2.005(5)
Zn(1)-O(12)	2.008(3)		Zn(2)-O(6)		2.010(3)
Zn(1)-N(2)	2.072(4)		Zn(2)-O(6)		2.054(4)
**Bond Angles (°)**					
O(1)-Zn(1)-O(4)	129.1(1)		O(7)-Zn(2)-O(10)#1		106.6(2)
O(1)-Zn(1)-O(12)	101.9(1)		O(7)-Zn(2)-O(6)		101.6(1)
O(4)-Zn(1)-O(12)	116.7(1)		O(10)#1-Zn(2)-O(6)		103.8(1)
O(1)-Zn(1)-N(2)	116.0(2)		O(7)-Zn(2)-N(7)		108.2(2)
O(4)-Zn(1)-N(2)	97.1(1)		O(10)#1-Zn(2)-N(7)		139.1(2)
O(12)-Zn(1)-N(2)	89.4(1)		O(6)-Zn(2)-N(7)		89.6(1)
**Intermolecular Interactions (Å)**					
**D-H···A**	**D-H (Å)**	**H···A (Å)**		**D···A (Å)**	**>D-H···A (°)**
N(1)-H(1)···O(2)	0.86	2.02		2.870(5)	173
N(4)-H(4)···O(8)	0.86	2.09		2.899(6)	157
C(46)-H(46A)···O(9)	0.96	2.53		3.429(7)	155
C(46)-H(46C)···O(8)	0.96	2.49		3.204(8)	131
C(31)-H(31)···Cg(4)	0.93	2.67		3.516(5)	151
C(40)-H(40)···Cg(4)	0.93	2.79		3.571(5)	142
C(64)-H(64)···Cg(1)	0.93	2.97		3.693(8)	135
#1: x + 1,y,z; Cg(1) = C(29) C(30) C(31) C(32) C(33) C(34); Cg(2) = N(7) C(19) C(47) C(48) C(49) C(50); Cg(3) = C(60) C(61) C(62) C(63) C(64) C(65); Cg(4) = C(51) C(52) C(53) C(54) C(55) C(56)					

**Table 2:** Selected bond lengths (Å), bond angles (°), and intermolecular interactions (Å) of compound **2**·4CH_3_CN.

Bond Lengths (Å)				
Zn(1)-O(1)	2.1028(15)		Zn(2)-O(2)	1.9465(14)
Zn(1)-O(4)	2.1644(15)		Zn(2)-O(8)	1.9673(15)
Zn(1)-O(7)	2.0861(15)		Zn(2)-O(5)	1.9254(15)
Zn(2)-N(4)	2.0358(17)			
**Bond Angles (°)**				
O(1)-Zn(1)-O(4)	90.39(6)		O(1)-Zn(1)-O(4)#1	89.61(6)
O(7)-Zn(1)-O(1)	90.00(6)		O(7)-Zn(1)-O(4)	93.10(6)
O(7)#1-Zn(1)-O(4)	86.90(6)		O(1)#1-Zn(1)-O(1)	180.00(7)
O(4)-Zn(1)-O(4)#1	180.00(4)		O(7)#1-Zn(1)-O(7)	180.0
O(2)-Zn(2)-N(4)	101.22(7)		O(5)-Zn(2)-N(4)	103.85(7)
O(8)-Zn(2)-N(4)	99.97(7)		O(5)-Zn(2)-O(2)	119.84(7)
O(2)-Zn(2)-O(8)	117.26(7)		O(5)-Zn(2)-O(8)	110.86(7)
**Twist Angles (°)**				
O(1)-Cg(1)-Cg(1)#1-O(4)	60.32		O(4)-Cg(1)-Cg(1)#1-O(7)	57.80
O(7)-Cg(1)-Cg(1)#1-O(1)	61.88			
**Intermolecular Interactions (Å)**				
**D-H···A**	**D-H (Å)**	**H···A (Å)**	**D···A (Å)**	**>D-H···A (°)**
C(44)-H(44)···N(1W)	0.95	2.60	3.496(4)	157
C(4W)-H(4W)A···O(9)	0.98	2.30	3.279(3)	177
C(4W)-H(4W)C···O(3)	0.98	2.46	3.428(4)	172
C(22)-H(22A)···Cg(2)	0.98	2.69	3.553(3)	148
C(27)-H(27)···Cg(3)	0.95	2.88	3.608(3)	134
Cg(3)···Cg(4)	3.8858(14)			
#1: -x + 1,-y + 1,-z + 1; Cg(1) = O(1) O(4) O(7); Cg(2) = C(26) C(27) C(28) C(29) C(30) C(31); Cg(3) = C(4) C(5) C(6) C(7) C(8) C(9); Cg(4) = N(4) C(34) C(35) C(36) C(37) C(38)				

**Table 3:** Selected bond lengths (Å), bond angles (°), and intermolecular interactions (Å) of compound **3**·4EtOH.

Bond Lengths (Å)					
Zn(1)-O(1)	2.172(3)		Zn(1)-O(4)		2.173(3)
Zn(1)-O(7)	2.159(3)		Zn(1)-O(10)		2.172(3)
Zn(1)-O(13)	2.150(3)		Zn(1)-O(16)		2.161(3)
Zn(2)-O(2)	1.952(2)		Zn(2)-O(5)		1.967(2)
Zn(2)-O(8)	1.934(2)		Zn(2)-O(19)		1.985(2)
Zn(3)-O(11)	1.9617(18)		Zn(3)-O(14)		1.940(2)
Zn(3)-O(17)	1.948(2)		Zn(3)-O(20)		1.985(2)
**Bond Angles (°)**					
O(1)-Zn(1)-O(4)	87.06(9)		O(7)-Zn(1)-O(1)		89.52(9)
O(7)-Zn(1)-O(4)	90.31(9)		O(7)-Zn(1)-O(10)		90.45(9)
O(7)-Zn(1)-O(16)	89.71(9)		O(10)-Zn(1)-O(4)		92.96(9)
O(13)-Zn(1)-O(1)	89.69(9)		O(13)-Zn(1)-O(4)		89.75(9)
O(13)-Zn(1)-O(10)	90.35(9)		O(13)-Zn(1)-O(16)		90.23(9)
O(16)-Zn(1)-O(1)	92.74(9)		O(16)-Zn(1)-O(10)		87.23(8)
O(1)-Zn(1)-O(10)	179.96(11)		O(13)-Zn(1)-O(7)		179.20(10)
O(16)-Zn(1)-O(4)	179.80 (11)		O(2)-Zn(2)-O(5)		114.81(10)
O(2)-Zn(2)-O(19)	99.26(11)		O(5)-Zn(2)-O(19)		105.43(11)
O(8)-Zn(2)-O(2)	122.91(10)		O(8)-Zn(2)-O(5)		115.08(9)
O(8)-Zn(2)-O(19)	92.69(10)		O(11)-Zn(3)-O(20)		99.53(10)
O(14)-Zn(3)-O(11)	123.27(10)		O(14)-Zn(3)-O(17)		113.34(10)
O(14)-Zn(3)-O(20)	97.72(10)		O(17)-Zn(3)-O(11)		115.20(9)
O(17)-Zn(3)-O(20)	107.00(10)				
**Twist Angles (°)**					
O(1)-Cg(1)-Cg(2)-O(13)	59.10		O(7)-Cg(1)-Cg(2)-O(16)		58.88
O(4)-Cg(1)-Cg(2)-O(10)	61.49				
**Intermolecular Interactions (Å)**					
**D-H···A**	**D-H (Å)**	**H···A (Å)**		**D···A (Å)**	**>D-H···A (°)**
O(19)-H(19)O···O(3W)	0.845(19)	1.861(19)		2.536(4)	135.8(17)
O(20)-H(20)O···O(2W)	0.83(2)	1.76(2)		2.585(4)	174(4)
O(3W)-H(3WO)···O(4W)	0.84	1.91		2.669(4)	149
O(2W)-H(2WO)···O(1W)	0.84	2.01		2.683(3)	136
O(4W)-H(4WO)···O(6)	0.84	1.85		2.685(4)	175
O(1W)-H(1WO)···O(18)	0.84	1.88		2.712(4)	172
C(31)-H(31)···O(4W)	0.95	2.54		3.481(5)	172
C(53)-H(53)···O(1W)	0.95	2.52		3.460(5)	172
C(4W)-H(4WA)···O(20)	0.98	2.61		3.418(7)	140
C(4W)-H(4WB)···O(2)	0.98	2.63		3.502(6)	148
C(7W)-H(7WB)···Cg(3)	0.99	2.97		3.954(7)	172
C(20)-H(20)···Cg(4)	0.95	2.88		3.674(4)	142
C(60)-H(60)···Cg(5)	0.95	2.90		3.705(4)	143
C(69)-H(69B)···Cg(4)	0.99	2.69		3.611(4)	155
C(67)-H(67A)···Cg(5)	0.99	2.85		3.703(4)	145
Cg(1) = O(1) O(4) O(7); Cg(2) = O(10) O(13) O(16); Cg(3) = C(15) C(16) C(17) C(18) C(19) C(20); Cg(4) = C(4) C(5) C(6) C(7) C(8) C(9); Cg(5) = C(37) C(38) C(39) C(40) C(41) C(42)					

**Table 4:** Comparison of the supramolecular interactions of compounds **2**·4CH_3_CN and **3**·4EtOH.

Complex	D-H···A	Number of Interactions	% Hirshfeld Surface Implied
**2**·4CH_3_CN	C-H···N_solvent_	2	6.0
	C-H···O_C=O_	4	7.9
	C-H···π	4	28.1
	π ··· π	4	2.9
**3**·4EtOH	O-H···O_solvent_	2	12.3
	O_C=O_···H-O_solvent_	2	
	C-H···O_solvent_	2	
	C-H_solvent_···O_COO_	1	
	C-H_solvent_···O_coordinated EtOH_	1	
	C-H··· π	5	20.4

**Table 5:** Crystal data and structure refinement for **1**, **2**·4CH_3_CN, and **3**·4EtOH.

	**1**	**2**·4CH_3_CN	**3**·4EtOH
Empirical Formula	C_66_H_58_N_6_O_12_Zn_2_	C_96_H_90_N_12_O_18_Zn_3_	C_78_H_96_N_6_O_24_Zn_3_
Formula weight	1257.92	1895.90	1697.71
T (K)	293(2)	100(2)	100(2)
Wavelength (Å)	0.71073	0.71073	0.71073
System, space group	Monoclinic, P2_1_/c	Monoclinic, P2_1_/c	Monoclinic, P2_1_
Unit cell dimensions			
a (Å)	10.1338(8)	15.7748(10)	13.2110(4)
b (Å)	24.469(2)	23.7227(15)	24.9706(9)
c (Å)	25.598(2)	13.1019(8)	13.3721(4)
β (°)	111.681(4)	112.111(2)	113.6880(10)
V (Å^3^)	5898.3(8)	4542.4(5)	4039.6(2)
Z	4	2	2
D_calc_ (mg/m^3^)	1.417	1.386	1.396
µ (mm^−1^)	0.883	0.861	0.962
F (000)	2608	1968	1776
Crystal size (mm^−3^)	0.141×0.052×0.032	0.150×0.368×0.398	0.142×0.252×0.505
hkl ranges	−11 <= h <= 14, −34 <= k <= 34, −36 <= l <= 36	−21 <= h <= 22, −33 <= k <= 33, −18 <= l <= 17	−19 <= h <= 19, −36 <= k <= 36, −19 <= l <= 19
2θ range (°)	2.176 to 30.705	2.211 to 30.554	2.452 to 30.972
Reflections collected/ unique/[R_int_]	80518/18035/ 0.2023	141282/13913/ 0.0604	25525/25525/ 0.0421
Completeness to θ (%)	98.4	99.8	99.7
Absorption correction	Semi-empirical	Semi-empirical	Semi-empirical
Max. and min. transmission	0.7461 and 0.6289	0.7461 and 0.6411	0.7461 and 0.6349
Refinement method	Full-matrix least-square on F^2^	Full-matrix least-square on F^2^	Full-matrix least-square on F^2^
Data/Restrains/Parameters	18035/0/780	13913/0/588	25525/17/1016
Goodness-on-fit on F^2^	1.038	1.043	1.032
Final R indices [I > 2σ(I)]	R_1_ = 0.0756 wR_2_ = 0.1335	R_1_ = 0.0442 wR_2_ = 0.1155	R_1_ = 0.0348 wR_2_ = 0.0823
R indices (all data)	R_1_ = 0.2135 wR_2_ = 0.1958	R_1_ = 0.0639 wR_2_ = 0.1259	R_1_ = 0.0473 wR_2_ = 0.0885
Extinction coefficient	n/a	n/a	n/a
Largest Diff. peak and hole (e. Å^−3^)	0.986 and −1.239	0.538 and −2.492	0.726 and −1.656

## Supplementary Materials

The following are available online at https://www.mdpi.com/1420-3049/25/16/3615/s1, Tables S1–S3. Intramolecular interactions of compounds **1**, **2**·4CH_3_CN, and **3**·4EtOH, Figure S1. HR-ESI-MS spectra of compound **1**; Figures S2–S4. FTIR-ATR spectra of compounds **1**–**3**; Figures S5–S7. 1H NMR spectra of compounds **1**–**3**; Figures S8–S10. 13C{^1^H} NMR and DEPT-135 spectra of compounds **1**–**3**; Figures S11 and S12. Hirshfeld surface d_norm_ and ESP representations of the co-crystallized solvent molecules in **2**·4CH_3_CN and **3**·4EtOH. Figures S13 and S14. Hirshfeld surface curvedness mappings with their corresponding ESP representations of **2**·4CH_3_CN and **3**·4EtOH; Figures S15–S17. Thermal ellipsoid plots of **1**, **2**·4CH_3_CN, and **3**·4EtOH. Complete information about the crystal structure and molecular geometry is available in. cif format as Supporting Information.
