# Supplementary material for: Construction of Zn(II) Linear Trinuclear Secondary Building Units from A Coordination Polymer Based on α-Acetamidocinnamic Acid and 4-Phenylpyridine
Source: Molecules. 2020 Aug 9;25(16):3615. doi: 10.3390/molecules25163615 (PMC7463536; doi:10.3390/molecules25163615)
Supplement: Supplementary file 1 [file molecules-25-03615-s001.pdf]

Supplementary Information

Construction of Zn(II) linear trinuclear secondary building units from a coordination polymer based on α-acetamidocinnamic acid and 4-phenylpyridine

Daniel Ejarque [1], Teresa Calvet [2], Mercè Font-Bardia [3] and Josefina Pons [1,*]

[1] Departament de Química, Universitat Autònoma de Barcelona, 08193-Bellaterra, Barcelona, Spain; daniel.ejarque@uab.cat (D.E.)

[2] Departament de Mineralogia, Petrologia i Geologia Aplicada, Universitat de Barcelona, Martí i Franquès s/n, 08028 Barcelona, Spain; mtcalvet@ub.edu (T.C.)

[3] Unitat de Difracció de Raig-X, Centres Científics i Tecnològics de la Universitat de Barcelona (CCiTUB), Universitat de Barcelona, Solé i Sabarís, 1-3, 08028 Barcelona, Spain; mercef@ccit.ub.edu (M.F.-B)

* Correspondence: josefina.pons@uab.es (J.P.); Tel.: +34-935-812-895

Intramolecular interactions

Table S1. Intramolecular interactions of compound [Zn2(μ-O,O′-ACA)$_2$(ACA)$_2$(4-Phpy)$_2$]$_n$ (**1**).

D-H···A	D-H (Å)	H···A (Å)	D···A (Å)	>D-H···A (°)
N(3)-H(3)···O(3)	0.86	1.88	2.742(5)	178
N(5)-H(5)···O(9)	0.86	1.88	2.737(6)	173
C(62)-H(62)···O(8)	0.93	2.34	3.121(6)	142
C(11)-H(11C)···Cg(1)	0.96	2.77	3.588(6)	144
Cg(2)···Cg(3)	3.745(3)			

Table S2. Intramolecular interactions of compound [Zn$_3$(μ-ACA)$_6$(4-Phpy)$_2$]·4CH$_3$CN (**2**·4CH$_3$CN)

D-H···A	D-H (Å)	H···A (Å)	D···A (Å)	>D-H···A (°)
N(1)-H(1)···O(9)	0.88	2.02	2.867(2)	162
N(2)-H(2)···O(3)	0.88	2.12	2.849(2)	140
N(3)-H(3)···O(6)	0.88	2.00	2.851(3)	

Table S3. Intramolecular interactions of compound [Zn$_3$(μ-ACA)$_6$(EtOH)$_2$]·4EtOH (**3**·4EtOH)

D-H···A	D-H (Å)	H···A (Å)	D···A (Å)	>D-H···A (°)
N(1)-H(1)···O(18)	0.88	2.06	2.912(4)	161
N(2)-H(2)···O(15)	0.88	2.00	2.854(4)	163
N(3)-H(3)···O(12)	0.88	2.10	2.875(3)	147
N(4)-H(4)···O(6)	0.88	2.05	2.902(4)	163
N(5)-H(5)···O(3)	0.88	2.07	2.872(3)	151
N(6)-H(6)···O(9)	0.88	2.01	2.850(4)	159

HR-ESI-MS

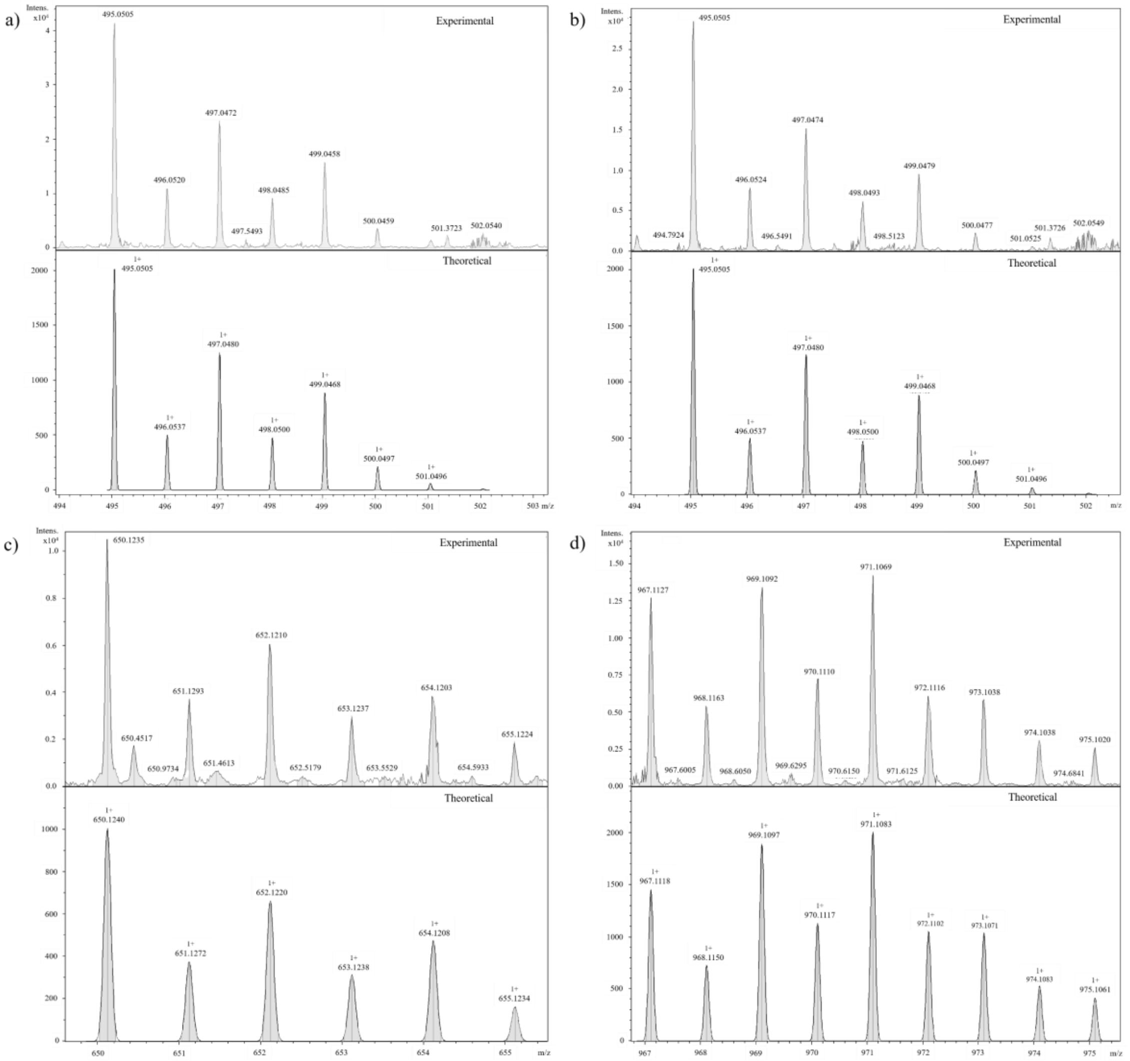

Figure S1. HR-ESI-MS spectra of compound $[Zn_2(\mu\text{-}O,O'\text{-}ACA)_2(ACA)_2(4\text{-}Phpy)_2]_n$ (**1**). In detail view of (**a**) $[Zn(ACA)_2 + Na]^+$ in CH_3CN. (**b**) $[Zn(ACA)_2 + Na]^+$ in EtOH. (**c**) $[Zn(ACA)_2(4\text{-}Phpy) + Na]^+$ in CH_3CN and (**d**) $[Zn_2(ACA)_4 + Na]^+$ in CH_3CN fragments.

FTIR-ATR, ^{1}H, ^{13}C{^{1}H} and DEPT-135 spectroscopies

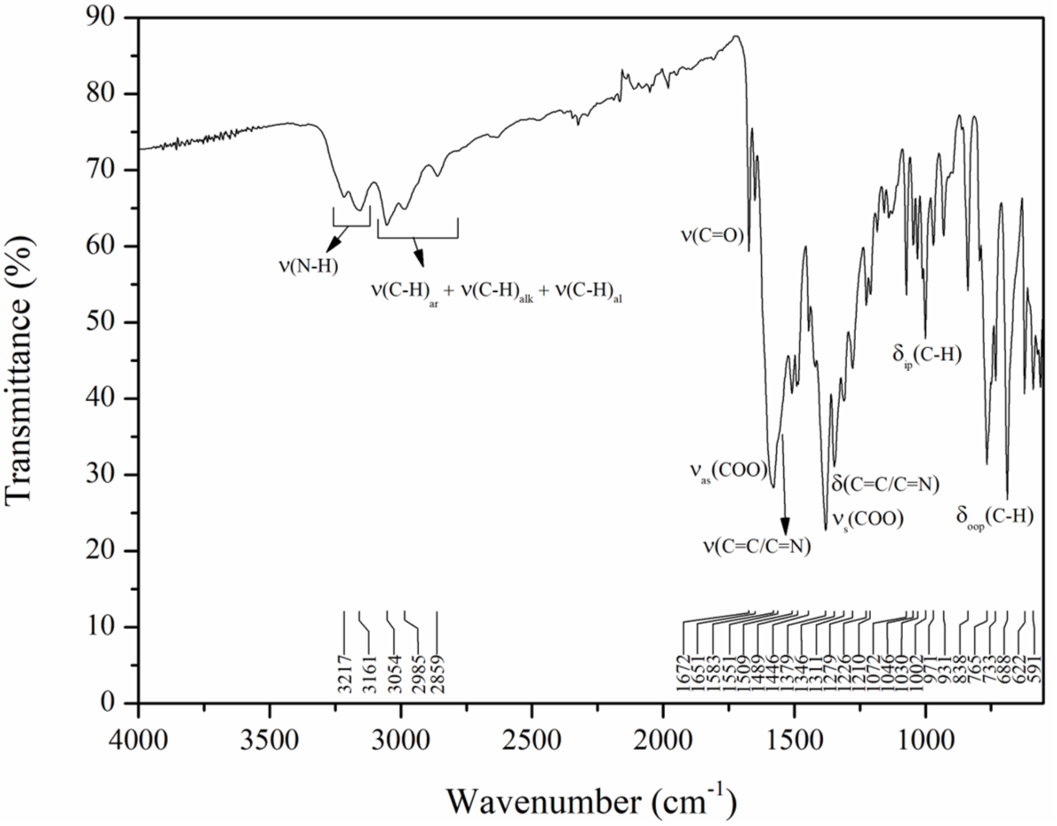

Figure S2. FTIR-ATR spectrum of compound $[Zn_2(\mu\text{-}O,O'\text{-}ACA)_2(ACA)_2(4\text{-}Phpy)_2]_n$ (**1**).

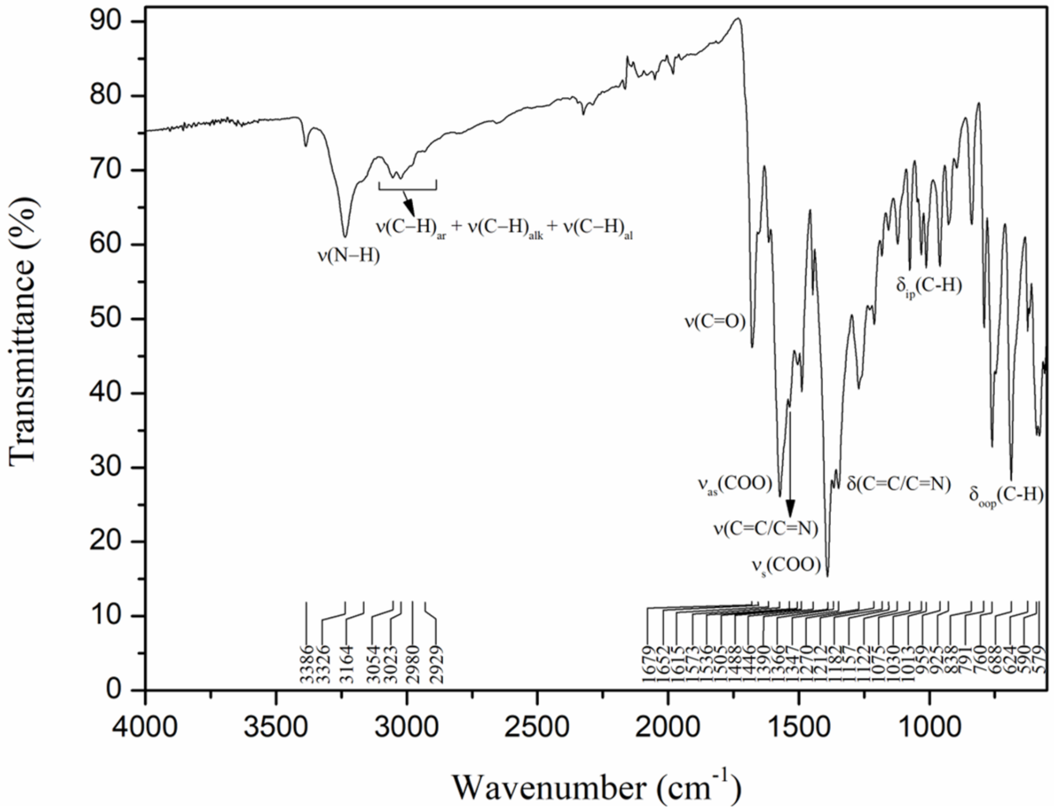

Figure S3. FTIR-ATR spectrum of compound $[Zn_3(\mu\text{-}ACA)_6(4\text{-}Phpy)_2]$ (**2**).

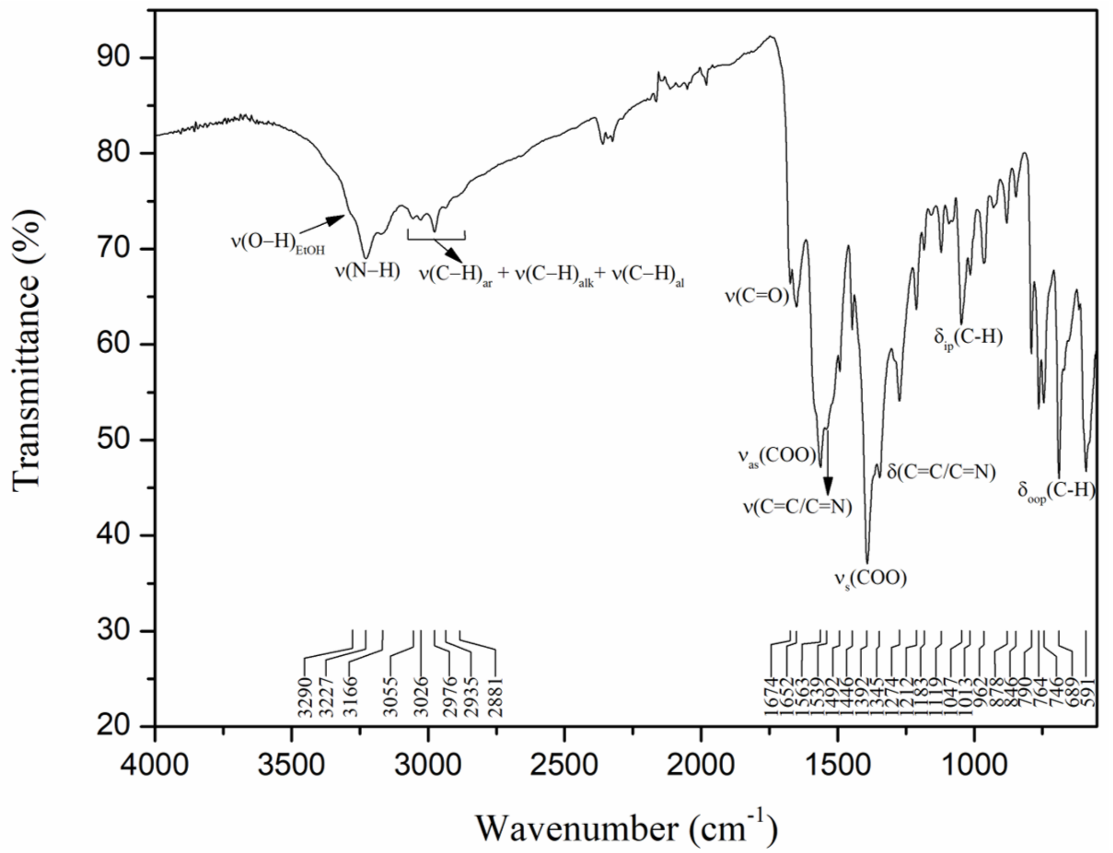

Figure S4. FTIR-ATR spectrum of compound [Zn$_3$(μ-ACA)$_6$(EtOH)$_2$] (**3**).

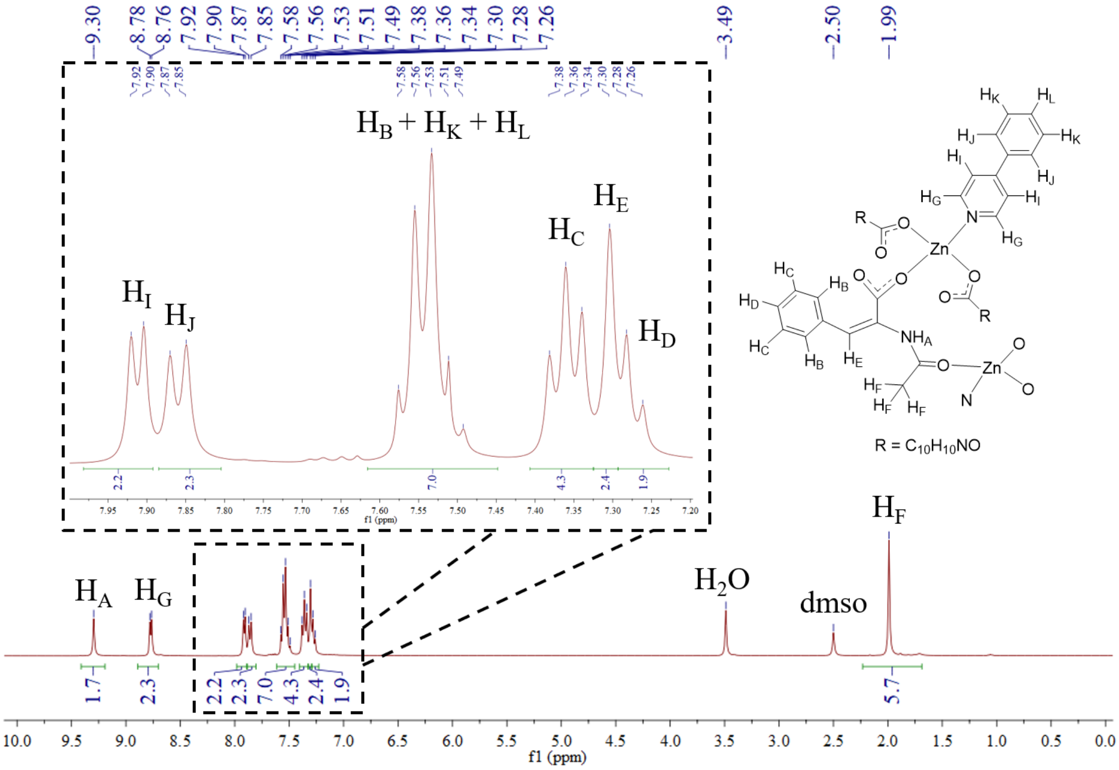

Figure S5. ^{1}H NMR spectrum of compound [Zn$_2$(μ-O,O′-ACA)$_2$(ACA)$_2$(4-Phpy)$_2$]$_n$ (**1**).

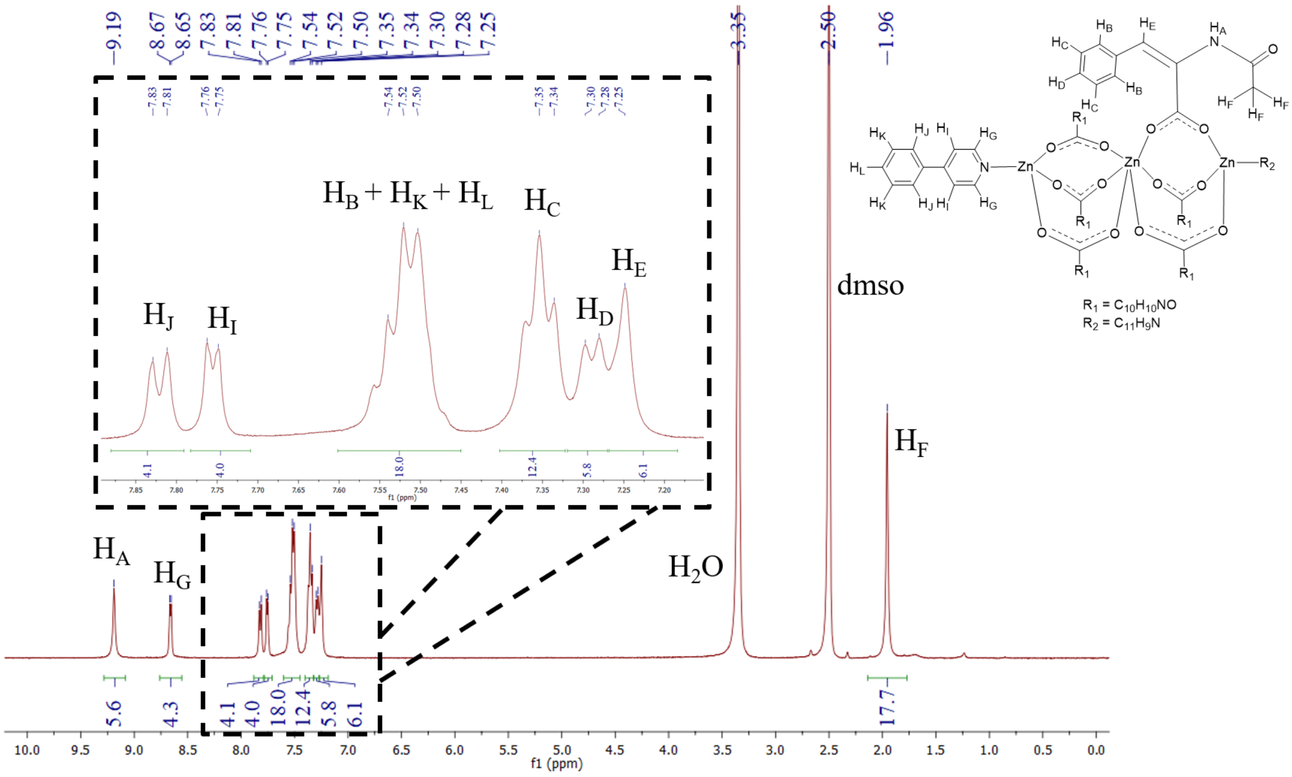

Figure S6. ^{1}H NMR spectrum of compound [Zn3(μ-ACA)6(4-Phpy)2] (**2**).

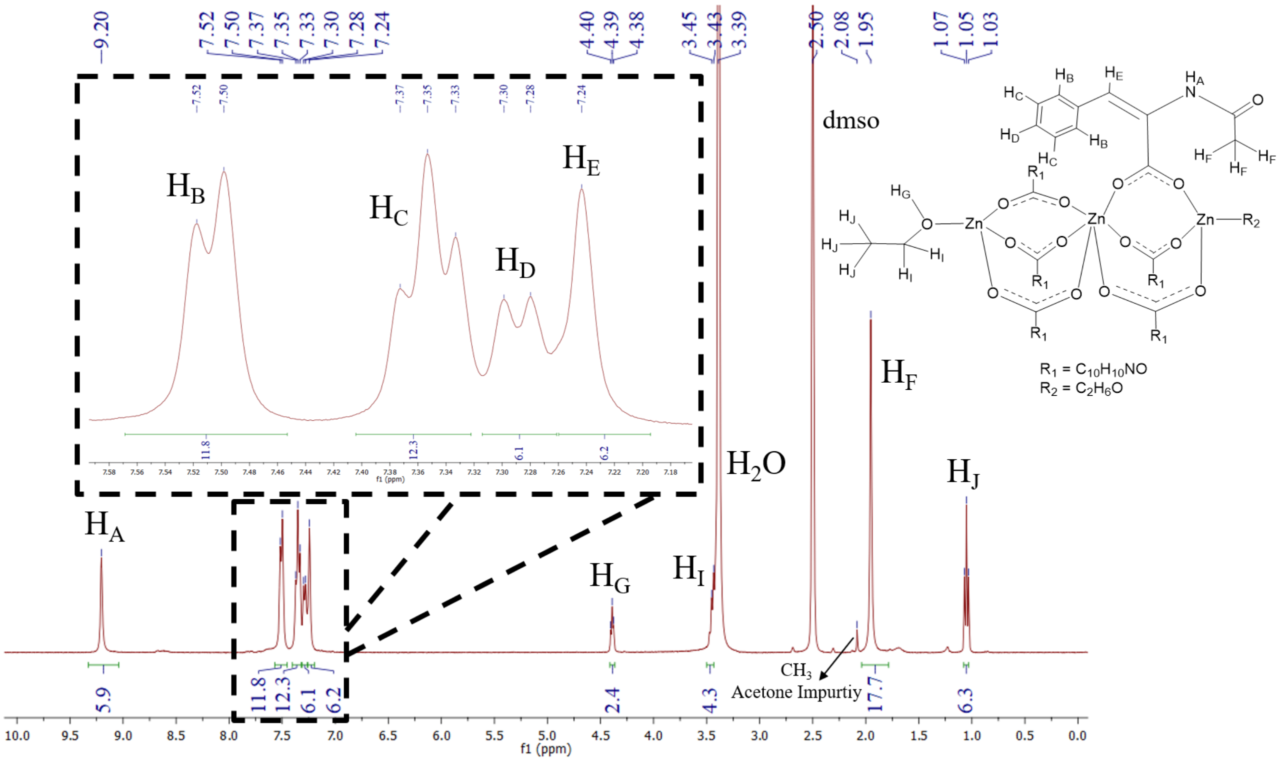

Figure S7. ^{1}H NMR spectrum of compound [Zn3(μ-ACA)6(EtOH)2] (**3**).

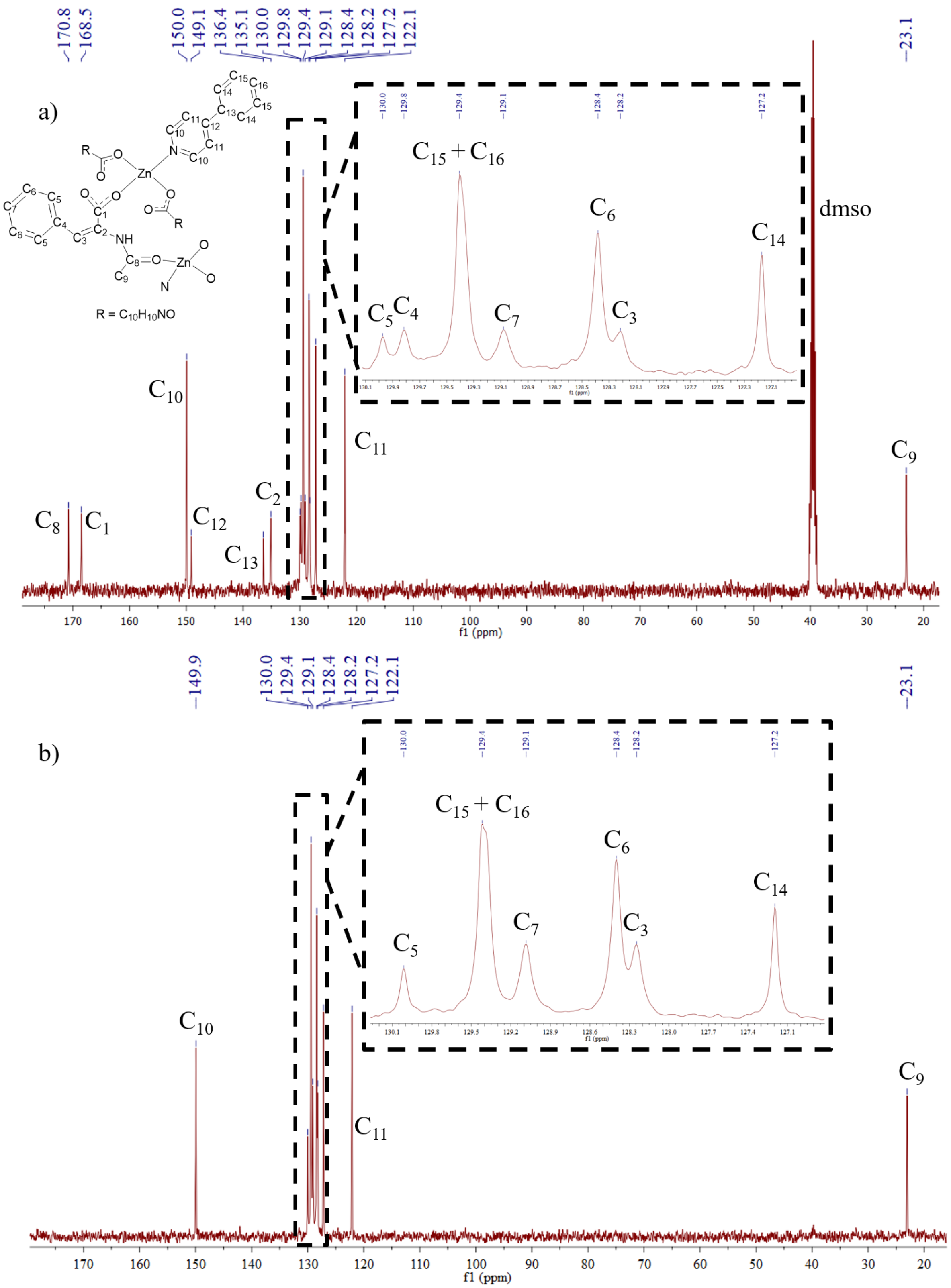

Figure S8. (**a**) $^{13}C\{^{1}H\}$ NMR spectrum and (**b**) DEPT-135 NMR spectrum of compound $[Zn_2(\mu\text{-}O,O'\text{-}ACA)_2(ACA)_2(4\text{-}Phpy)_2]_n$ (**1**).

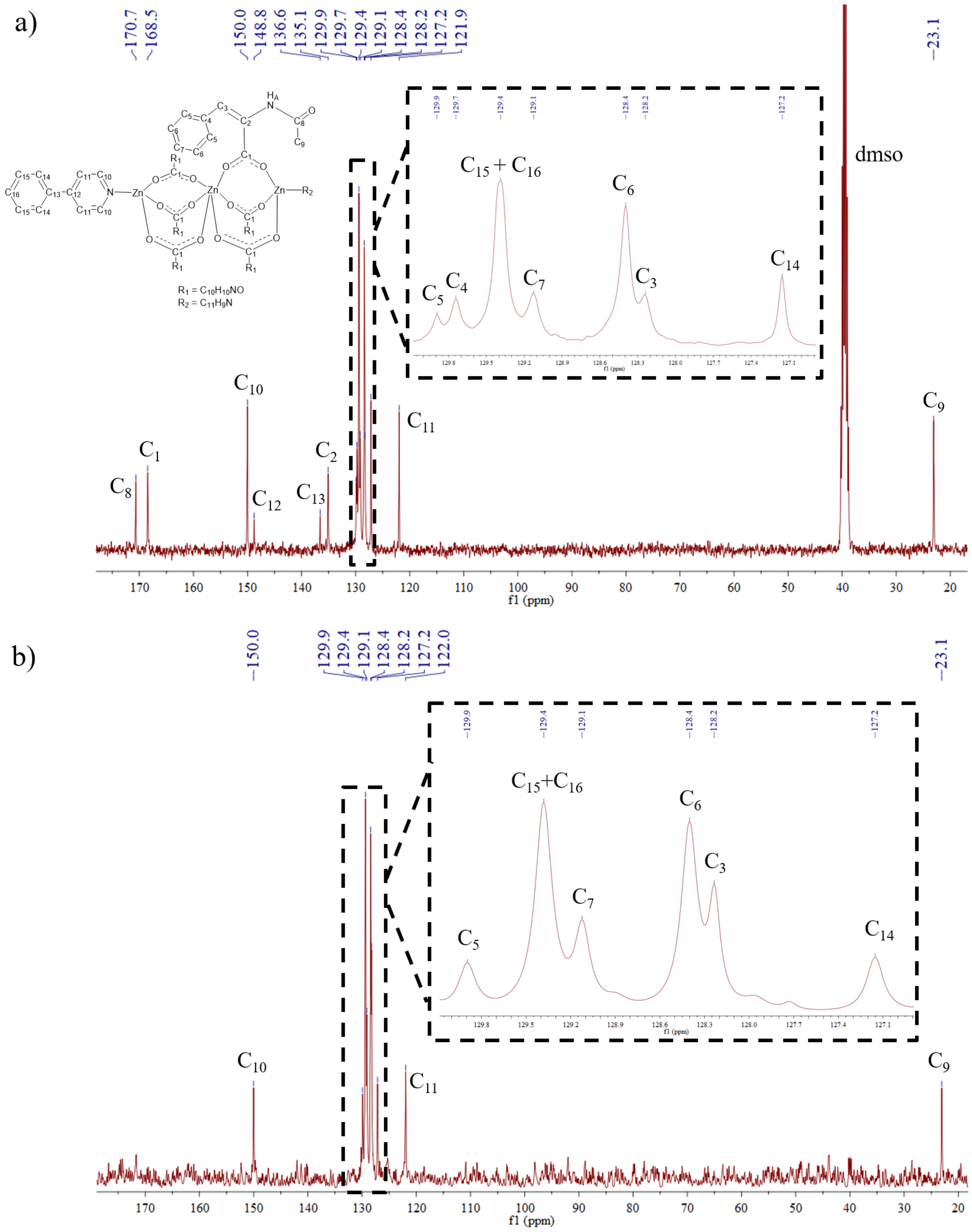

Figure S9. (**a**) $^{13}C\{^{1}H\}$ NMR spectrum and (**b**) DEPT-135 NMR spectrum of compound $[Zn_3(\mu\text{-}ACA)_6(4\text{-}Phpy)_2]$ (**2**).

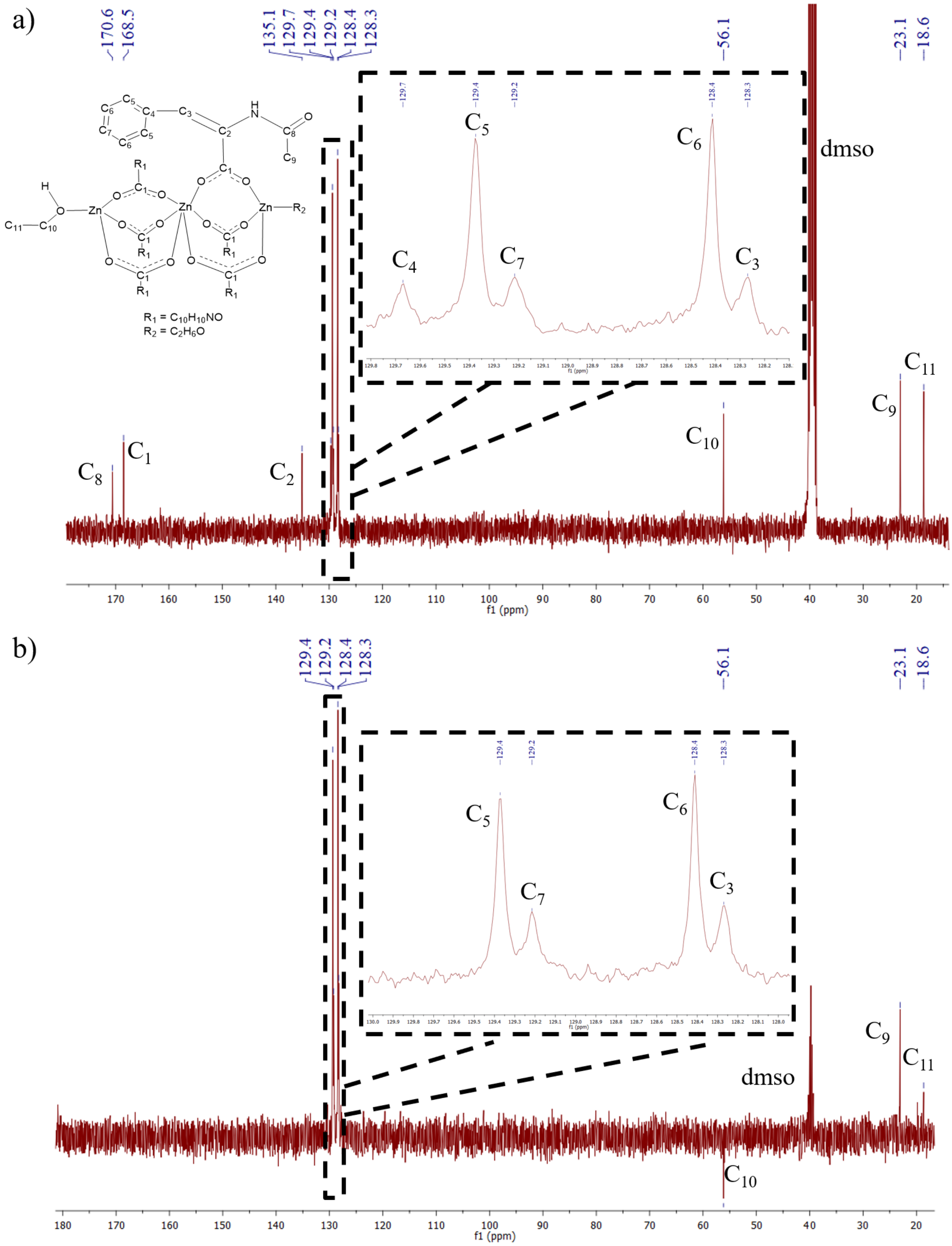

Figure S10. (**a**) $^{13}C\{^1H\}$ NMR spectrum and (**b**) DEPT-135 NMR spectrum of compound $[Zn_3(\mu\text{-}ACA)_6(EtOH)_2]$ (**3**).

Hirshfeld surface analysis

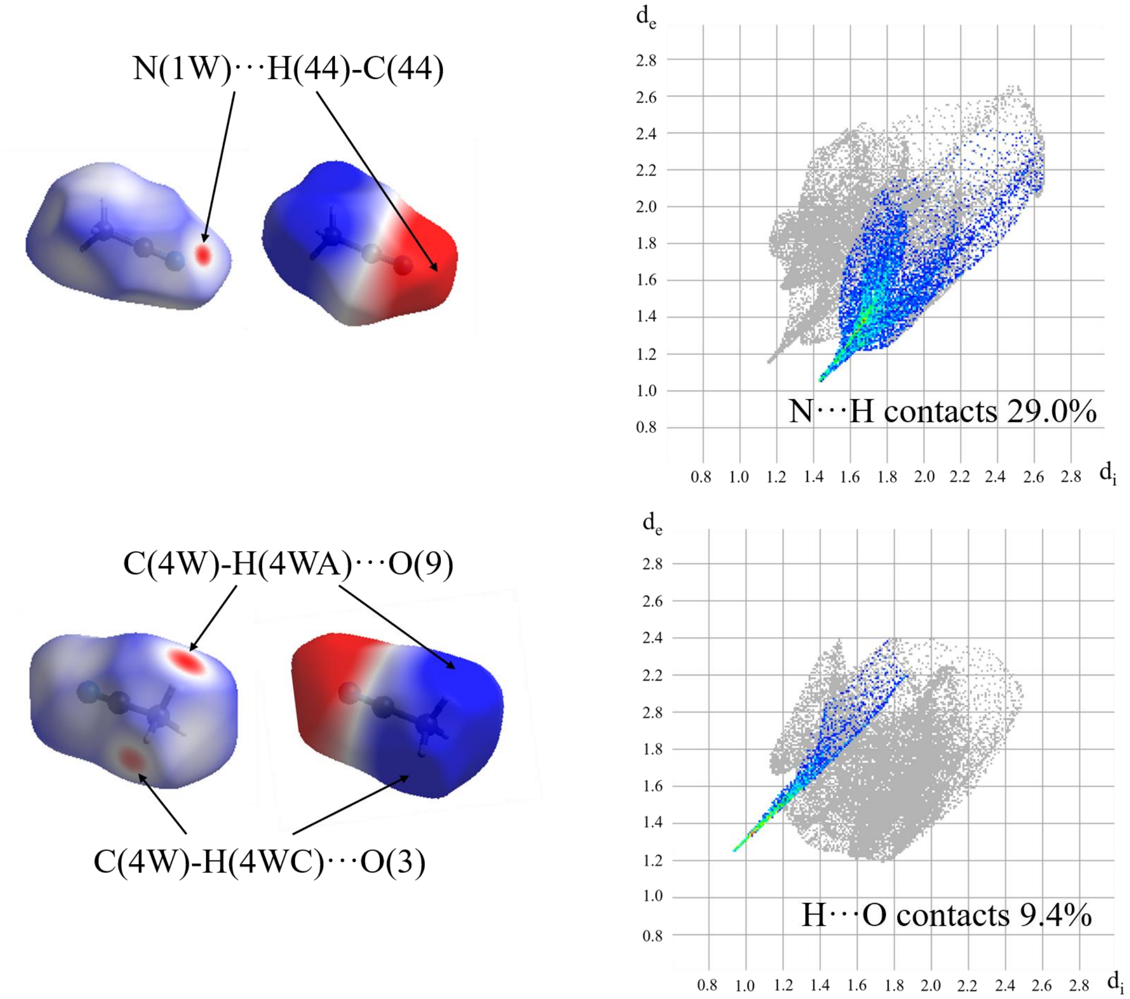

Figure S11. Hirshfeld surface d_{norm} (left mapping) and ESP (right mapping) representations of the co-crystallized CH_3CN molecules in **2**·4CH_3CN with their intermolecular interactions assigned and their corresponding regions of surface involved in the 2D fingerprint plot highlighted.

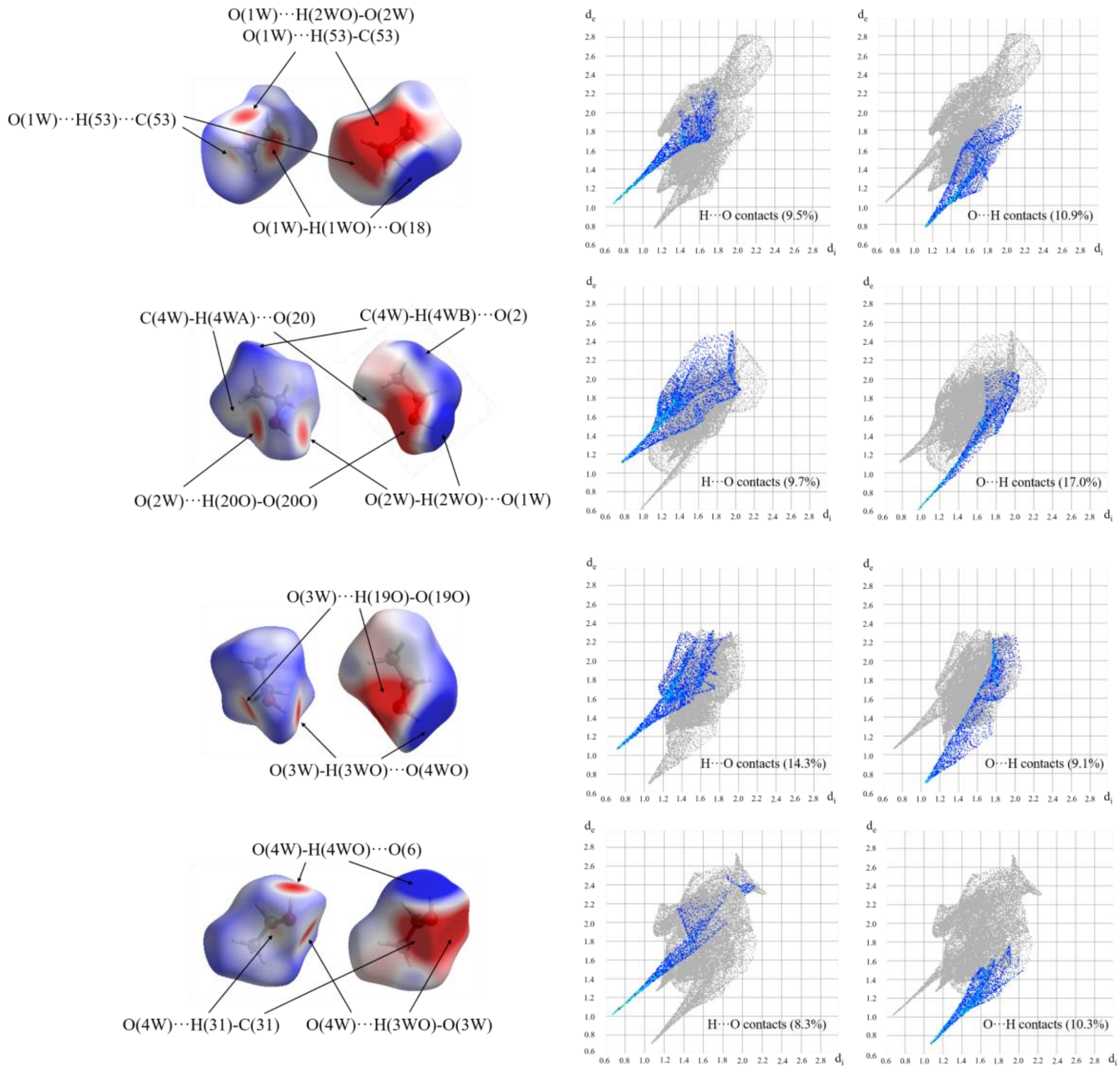

Figure S12. Hirshfeld surface d_{norm} (left mapping) and ESP (right mapping) representations of the co-crystallized EtOH molecules in structure **3**·4EtOH with their intermolecular interactions assigned and their corresponding regions of surface involved in the 2D fingerprint plot highlighted.

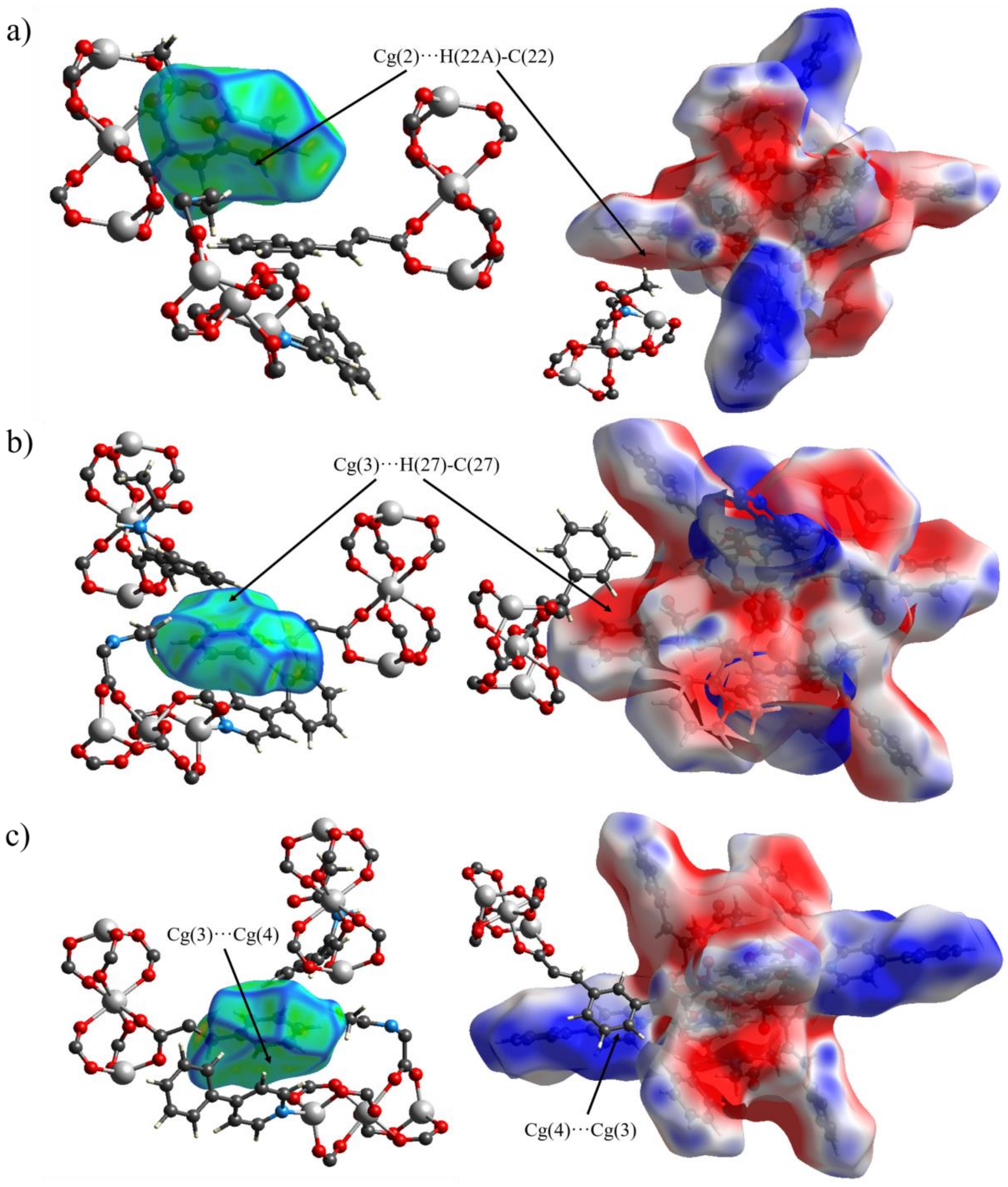

Figure S13. Hirshfeld surface curvedness mappings highlighting the planar interactions with their corresponding ESP representations of compound **2**·4CH_3CN involving (**a**) Cg(2)···H(22A)-C(22). (**b**) Cg(3)···H(27)-C(27) and (**c**) Cg(3)···Cg(4) interactions.

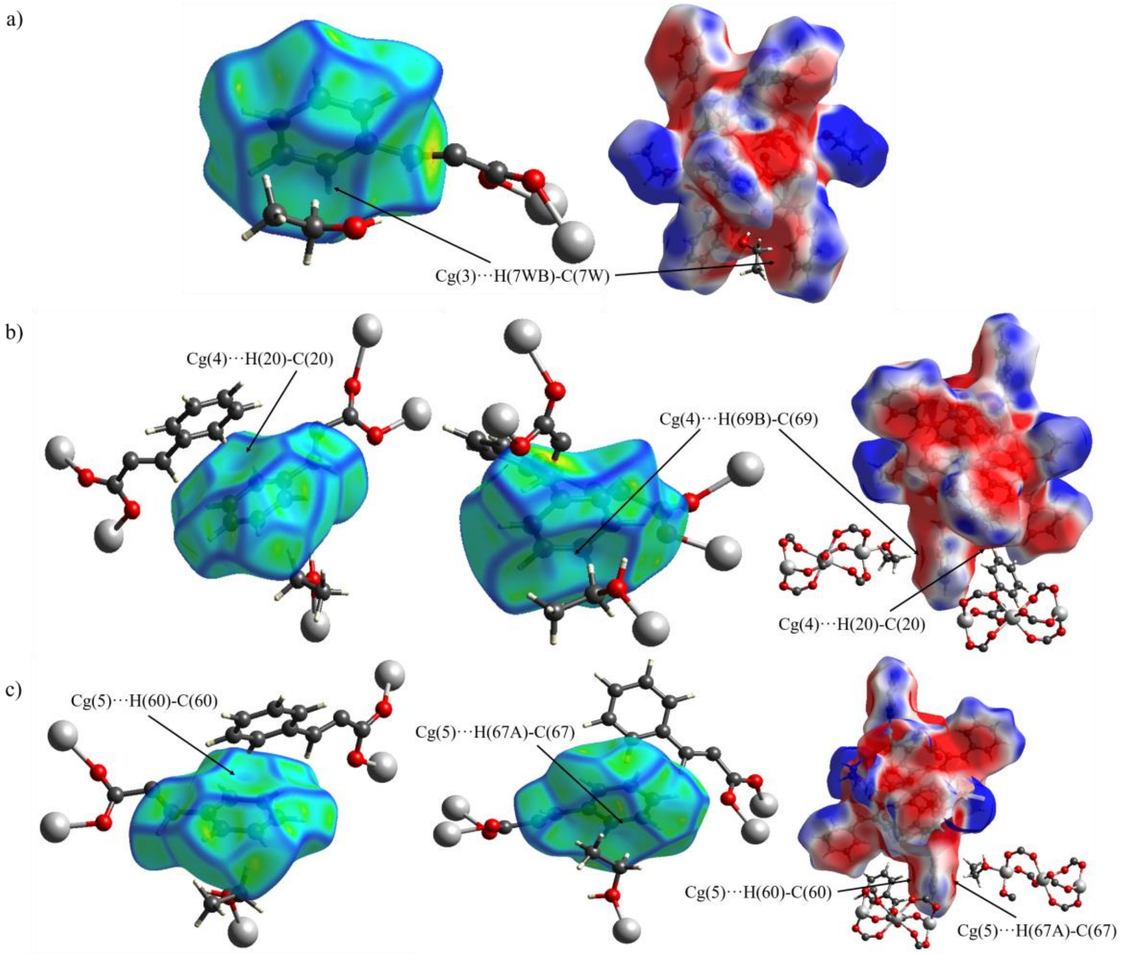

Figure S14. Hirshfeld surface curvedness mappings highlighting the planar interactions with their corresponding ESP representations of compound **3**·4EtOH involving (**a**) Cg(3). (**b**) Cg(4) and (**c**) Cg(5) centroids.

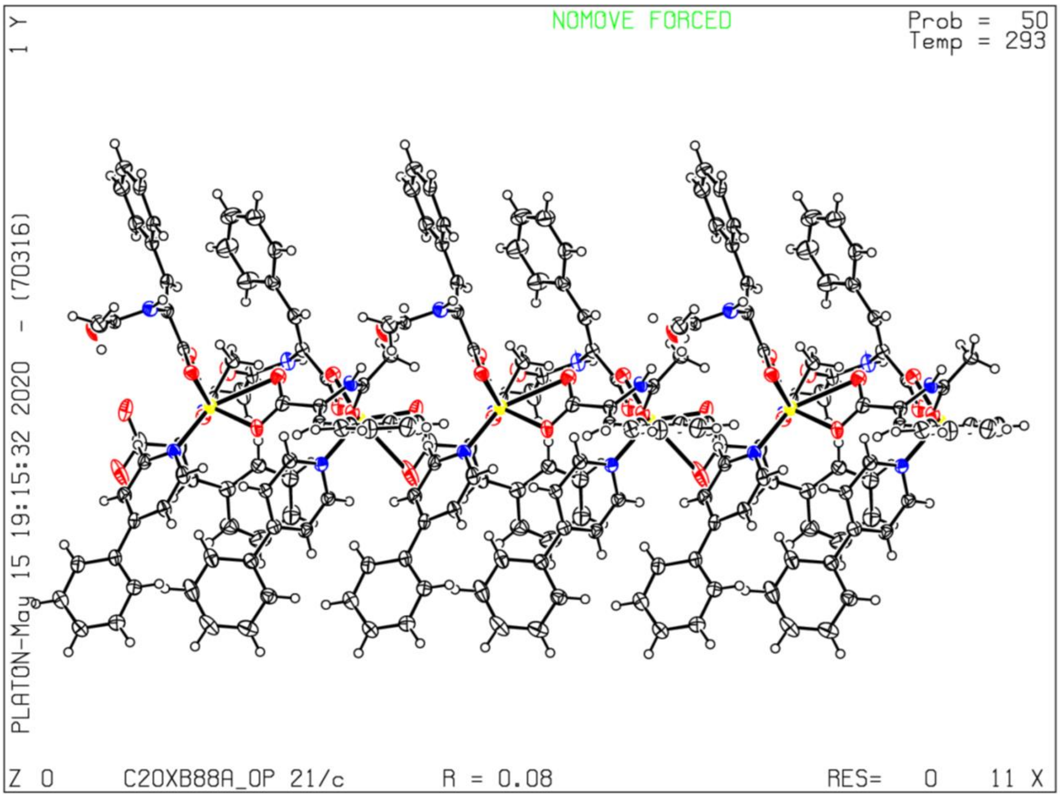

Figure S15. Thermal ellipsoid plot of structure [Zn_2(μ-O,O′-ACA)$_2$(ACA)$_2$(4-Phpy)$_2$]$_n$ (**1**).

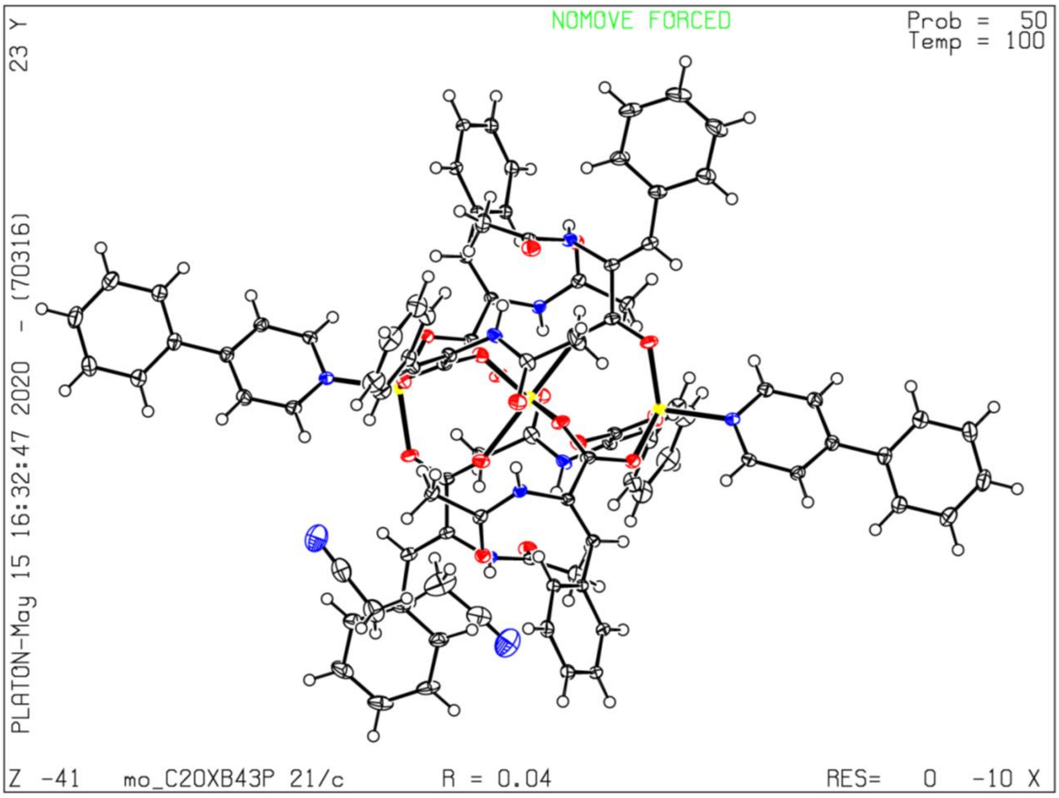

Figure S16. Thermal ellipsoid plot of structure [Zn_3(μ-ACA)$_6$(4-Phpy)$_2$]·4CH_3CN (**2**·4CH_3CN).

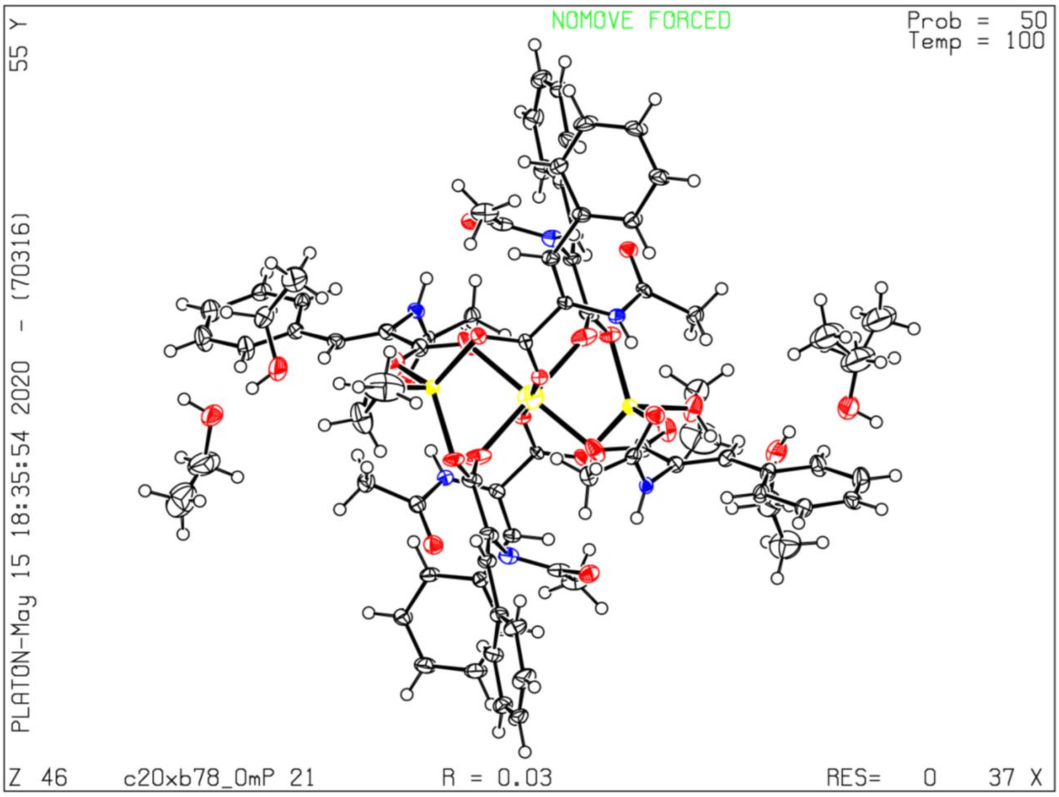

Figure S17. Thermal ellipsoid plot of structure [Zn_3(μ-ACA)$_6$(EtOH)$_2$]·4EtOH (**3**·4EtOH).
